# Liver fibrosis: Pathogenesis and innovative nanoparticle-based therapeutic strategies

**DOI:** 10.1016/j.ijpx.2026.100596

**Published:** 2026-07-01

**Authors:** Yibin Cai, Jiayin Li, Shenyu Chen, Hui Zhang, Jie Tan, Hao Li, Shuyuan Zhang, Boran Li, Qin Wei, Chenshi Lin, Xuesong Zhong, Xiuhua You, Chao Qin, Chao Teng, Jie Liu

**Affiliations:** a Department of Thoracic Surgery, Clinical Oncology School of Fujian Medical University, Fujian Cancer Hospital, Fuzhou 350014, China; b National Engineering Laboratory for AIDS Vaccine, School of Life Sciences, Jilin University, Changchun 130012, China; c Faculty of Medicine, The University of Queensland, Brisbane, Australia; d School of Pharmacy, Fujian Medical university, Fuzhou 350122, China; e Fujian Key Laboratory of Natural Medicine Pharmacology, School of Pharmacy, Fujian Medical University, Fuzhou 350122, China; f School of Pharmacy, China Pharmaceutical University, Nanjing 210009, China; g Department of Pharmacy, Jiujiang Traditional Chinese Medicine Hospital Affiliated to Jiangxi University of Traditional Chinese Medicine, Jiujiang 332000, China; h School of Business, China Pharmaceutical University, Nanjing 210009, China

**Keywords:** Liver fibrosis, Hepatic stellate cells, Extracellular matrix, Targeted delivery

## Abstract

Liver fibrosis is a dynamic pathological process characterized by excessive extracellular matrix (ECM) deposition, primarily driven by the activation of hepatic stellate cells (HSCs). Recent advances in liver fibrosis research have identified critical pathways, such as oxidative stress, inflammation and transforming growth factor-β (TGF-β) signaling, which drive disease progression. Concurrently, innovative drug delivery systems (e.g., liposomes, micelles and nanocrystals) have emerged to enhance therapeutic efficacy and targeting. These systems enable precise delivery of antifibrotic agents to HSCs or fibrotic tissues, minimizing off-target effects and improving pharmacokinetic profiles. This review summarizes current understanding of liver fibrosis pathogenesis and recent advances in drug delivery strategies, highlighting clinical transformation opportunities and future research directions. Combining molecular insights with advanced delivery technologies represents a promising avenue for developing effective antifibrotic therapies.

## Graphical abstract

Scheme. Mechanisms of liver fibrosis and advancements in drug delivery strategies. A detailed exploration of the underlying mechanisms driving liver fibrosis, alongside cutting-edge advancements in drug delivery systems, highlighting their potential to improve therapeutic efficacy in combating the diseases.

**Figure:**
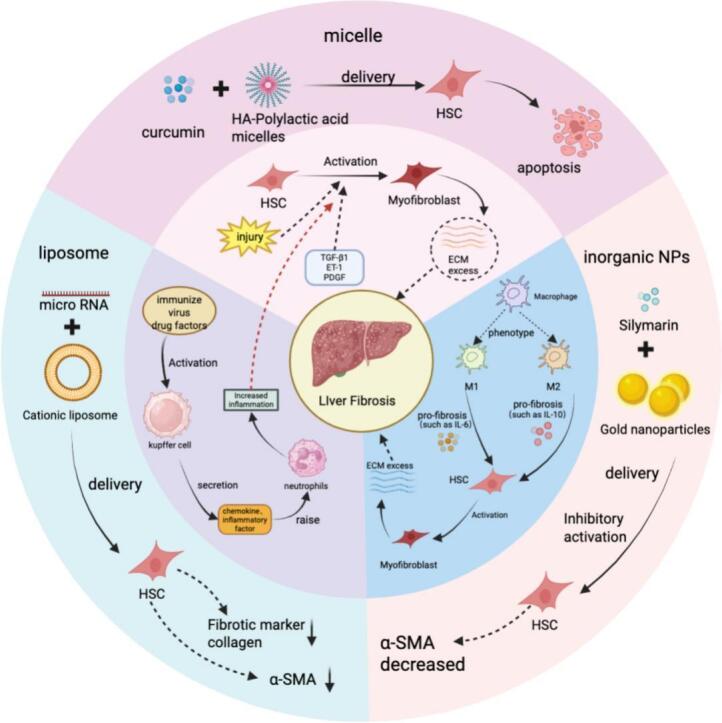

## 1 Introduction

Liver disease poses a major threat to human health worldwide, causing approximately 2 million deaths each year (Gan et al., 2025; Devarbhavi et al., 2023). Among them, liver fibrosis remains a major burden on global health systems. Non-alcoholic fatty liver disease and non-alcoholic steatohepatitis are considered to be the main causes of liver fibrosis (Levada et al., 2019; Le et al., 2024). This condition arises from chronic liver damage combining with extracellular matrix (ECM) deposition (Bataller and Brenner, 2005). Persistent damage triggers a pro-inflammatory response, disrupting the normal liver architecture and physiological function. This process may then progress to cirrhosis, portal hypertension or even liver cancer. Although current reports indicate that early liver fibrosis is reversible, the specific mechanism of reversing liver fibrosis is unclear, and effective liver fibrosis treatments are still lacking. Therefore, a better understanding of the cellular pathways of fibrosis could clarify our understanding of the process and uncover potential therapeutic targets. Current research focuses on four major strategies, anti-inflammatory therapy, inhibition of hepatic stellate cells (HSCs) activation and proliferation, reduction of excessive ECM production, and acceleration of ECM degradation. Here, we summarize the latest research progress on the pathological mechanism and therapeutic strategies of liver fibrosis, and discuss the future prospects in the field of liver fibrosis treatment.

## 2 Pathogenesis

### 2.1 Overview of liver fibrosis pathogenesis

**Fig. 1:**
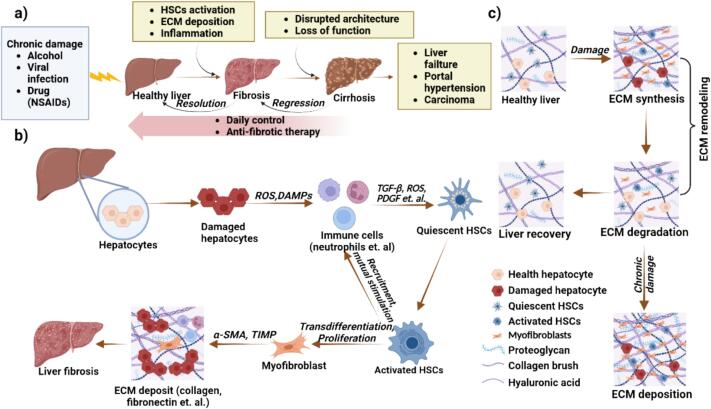
Overview of liver fibrosis pathogenesis. (a) The development of liver fibrosis. Chronic liver injury (such as alcohol, viral infection, nonsteroidal anti-Inflammatory drugs, *etc.*) can trigger the activation of hepatic stellate cells (HSCs), extracellular matrix (ECM) deposition and inflammatory responses, ultimately leading to fibrosis and liver cirrhosis. (b) Hepatocyte injury induces liver fibrosis. Damaged hepatocytes release reactive oxygen species (ROS) and damage-associated molecular patterns (DAMPs), promoting the activation and transdifferentiation of HSCs into myofibroblasts, which secrete large amounts of ECM components, leading to liver fibrosis. (c) The mechanisms of ECM remodeling. During the ECM reconstruction process, in the early stage, the synthesis of ECM increased due to tissue damage. With the persistent of chronic damage, a large amount of ECM deposition will occur.

Liver fibrosis is a pathological process caused by various chronic liver damage factors, involving viral infections, alcohol consumption, and certain drug toxicity. It is manifested as excessive accumulation of ECM, particularly collagen fibers, leading to hepatic structural remodeling and functional impairment. Clinically, liver fibrosis is often marked by impaired liver function and metabolic dysregulation (Steinberg et al., 2025; Lau and Hu, 2024). The pathogenesis of liver fibrosis involves initial liver damage, hepatic inflammation and dysfunction (Fig. 1). Firstly, extrinsic factors such as viral infections, alcohol consumption, and certain medications can cause initial liver damage which continuously occurs and eventually induces an inflammatory response (Seitz et al., 2018; Andrade et al., 2019; Woolbright and Jaeschke, 2018; Khatun and Ray, 2019). During this inflammatory response, certain hepatocytes become activated, secrete copious collagen fibers and other ECM proteins, culminating in connective tissue deposition within the liver (Lua et al., 2016; Yang et al., 2019b). Consequently, there is a progressive loss of function and disrupted architecture in the liver. Liver fibrosis is reversible in the early stage upon elimination of the underlying cause, but without timely intervention, it may irreversibly develop into cirrhosis. Thus, early detection and intervention in liver fibrosis are crucial to halt its progression to cirrhosis.

### 2.2 Hepatic stellate cells (HSCs) activation and fibrogenesis

The activation of HSCs is pivotal in the progression of liver fibrosis. In healthy livers, HSCs are mainly responsible for storing vitamin A (VA) and exist in a quiescent state. However, when the liver is chronically damaged, quiescent HSCs can be activated, which is characterized by prolific secretion of ECM components (primary constituents of fibrotic tissue) (Tsuchida and Friedman, 2017b). This transition results in significant alterations in liver architecture, including reduction of normal hepatocytes and vascular remodeling, even the progression of cirrhosis (Kisseleva and Brenner, 2021a; Lin et al., 2022b; Hegazy Rabab, 2025). Therefore, it is evident that activated HSCs play a critical role in exacerbating liver fibrosis through the overproduction of ECM components.

Moreover, the exacerbation of liver fibrosis by activated HSCs is further influenced by specific cytokines released in the liver microenvironment. Some cytokines, particularly transforming growth factor β (TGF-β) and platelet-derived growth factor (PDGF), are secreted by damaged hepatocytes and immune cells, directly modulate HSCs activity (Chen et al., 2026; Yan et al., 2025). TGF-β, in particular, has been demonstrated to enhance the proliferation of activated HSCs and stimulate them to secrete increased amounts of ECM components into the liver microenvironment, thereby potentially accelerating fibrotic progression (Dewidar et al., 2019; Wilhelm et al., 2016). PDGF, another cytokine in liver fibrosis, acts as a potent mitogen that prompts extensive proliferation and accumulation of HSCs in the inflammatory damaged areas. Furthermore, PDGF induces HSCs to produce additional cytokines, including TGF-β, which further sustain the increase of HSCs proliferation and ECM component secretion (Wilhelm et al., 2016). Consequently, hepatic injury triggers a cascade of signaling molecules from HSCs and other cells, expanding the activated HSCs population and amplifying ECM deposition, which contribute to the exacerbation of liver fibrosis.

Except for secreted cytokines, accumulated reactive oxygen species (ROS) in the inflammatory liver microenvironment also modulate the activity of HSCs. Previous studies have clarified that elevated ROS levels facilitate the activation of key transcription factors in HSCs, such as nuclear factor-kappa B (NF-κB), through oxidative modification. This activation perpetuates HSCs activity, leading to increased secretion of ECM components under oxidative stress (Ramos-Tovar and Muriel, 2020). In addition, ROS are implicated in the induction of the transcription factor activator protein-1 (AP-1), which augments the expression of tissue inhibitor of matrix metalloproteinases 1 (TIMP-1) and interleukin-6 (IL-6) in HSCs (Seki and Schwabe, 2015). Elevated TIMP-1 suppresses ECM degradation, thereby exacerbating fibrotic accumulation (Arpino et al., 2015; Lachowski et al., 2019). Simultaneously, higher secretion of IL-6 can promote inflammatory reaction in the lesion area (Schmidt-Arras and Rose-John, 2016; Deng et al., 2026). These findings collectively emphasize the significant role of ROS in improving both ECM deposition and inflammatory response in the liver through modulating HSCs activity.

The loss of VA-containing lipid droplets is a prominent feature of HSCs activation. Following hepatic injury, TGF-β induces the expression of matrix metalloproteinases (MMPs) in HSCs, thus facilitating the breakdown of VA-containing lipid droplets (Roeb, 2018; Kamm and Mccommis, 2022). At the same time, ROS can trigger lipid peroxidation by directly attacking unsaturated fatty acids in lipid droplets, leading to leakage and degradation of lipid droplet contents (Mooli et al., 2022). All these indicated that liver damage can enhance MMPs activity and ROS levels, which in turn contributes to the depletion of lipid droplets in HSCs, where VA is stored. This loss heralds a transition of HSCs to a myofibroblast phenotype with fibrogenic properties (Bobowski-Gerard et al., 2018; Kisseleva and Brenner, 2021a). During this transformation, the expression of α-smooth muscle actin (α-SMA) was markedly upregulated in these myofibroblasts. Previous studies have shown that increased expression of α-SMA correlates with enhanced synthesis and secretion of ECM components, further exacerbating liver fibrosis (Foglia et al., 2019; Munsterman I et al., 2018; Shang et al., 2018; Zhang et al., 2026a). Consequently, α-SMA serves not only as a biomarker for HSCs activation but also as a critical indicator of liver fibrosis progression. Targeting α-SMA expression or its upstream signaling pathways emerges as a potential therapeutic strategy in the management of liver fibrosis.

### 2.3 Inflammatory mediators and immune activation

When the liver is damaged, apoptotic hepatocytes release various damage-associated molecular patterns (DAMPs), such as high mobility group box-1 (HMGB1) protein and extracellular histones. These DAMPs play a pivotal role in activating Toll-like receptors (TLRs), particularly TLR4 and TLR9, which are crucial in initiating immune responses (Raza et al., 2025; Dong et al., 2026; Gupta and Vadde, 2025). TLRs, as pattern recognition receptors, mobilize innate immune cells by binding to their ligands, thereby promoting the infiltration of inflammatory cells and initiating immune responses. (Kim and Seki, 2020).

The infiltration and accumulation of some immune cells, such as neutrophils, T cells, B cells, macrophages and Kupffer cells (KCs), can lead to the release of various pro-inflammatory mediators in the liver microenvironment (Hammerich and Tacke, 2023b; Dixon et al., 2013; Liu et al., 2021; Krenkel et al., 2018). Secreted pro-inflammatory cytokines such as tumor necrosis factor-α (TNF-α), interleukin-1 (IL-1) and IL-6 are instrumental in the early stages of the inflammatory response. These cytokines promote immune cell recruitment and activation, as well as intercellular interactions, thereby amplifying the inflammatory milieu, which contributes to the development of liver fibrosis (Dixon et al., 2013; Hammerich and Tacke, 2023b; Niederreiter and Tilg, 2018).

KCs are a special type of immune cell in the liver that is primarily responsible for pathogen removal and liver metabolism (Araujo David et al., 2026). They secrete TGF-β, a multifunctional cytokine significant in activating HSCs (Gandhi, 2017). Once activated, HSCs transform into myofibroblasts upon activation and release ECM components, leading to liver fibrosis (Tsuchida and Friedman, 2017b). Therefore, KCs can deteriorate liver fibrosis through cellular crosstalk with HSCs. Additionally, neutrophils release significant amounts of ROS at inflammation sites, which further activate and proliferate HSCs (Cho and Szabo, 2021; Zhang et al., 2017; Yang et al., 2019b). These results indicated that neutrophils may exacerbate liver fibrosis through enhanced ROS levels.

Under chronic inflammation, persistent pro-inflammatory mediator secretion and ECM component accumulation creates a vicious cycle, leading to irreversible fibrosis progression, liver architectural distortion, and functional decline (Ebrahimi et al., 2018; Kisseleva and Brenner, 2021a). Given these dynamics, understanding the interplay of inflammatory mediators and immune activation in the progression of liver fibrosis is critical for developing novel therapeutic strategies. Future research should focus on effectively modulating these inflammatory mediators, arresting the progression of liver fibrosis, and exploring avenues to reverse fibrous tissue that has formed and restore normal liver function.

### 2.4 ECM Remodeling

ECM is an intricate network of proteins and polysaccharides that form a scaffold providing structural and biochemical support to the liver. Key ECM components include collagen, elastin, and glycoproteins (Manou et al., 2019; Baiocchini et al., 2016). Collagen is synthesized in its precursor form, known as procollagen. The procollagen undergoes folding and modification within the endoplasmic reticulum, followed by further modification in the Golgi apparatus. Once modified, procollagen can be secreted into the extracellular space, where it is transformed into mature collagen fibers by collagenases (Nimni and Harkness, 2018; Arseni et al., 2018; Roderfeld, 2018). The collagen fibers, along with other ECM components, undergo cross-linking in the extracellular environment and form a stable structure. Normally, cells interact with the ECM through specific surface receptors, influencing cellular migration, proliferation, and differentiation (Yamada and Sixt, 2019; Duarte et al., 2015; Roderfeld, 2018).

ECM remodeling is a critical process in maintaining the structure and function of liver, characterized by a delicate balance between ECM components synthesis and degradation. Under physiological conditions, ECM remodeling serves as a homeostatic mechanism vital for maintaining liver integrity and functionality (Duarte et al., 2015; Michalopoulos, 2017). Following temporary liver damage, activated HSCs upregulate the production of ECM components within the liver microenvironment, as part of the repair process (Roderfeld, 2018; Parola and Pinzani, 2019). ECM components, notably fibronectin and laminin, can interact with integrins on the surface of cells, triggering intracellular signaling cascades such as the tyrosine kinase pathway. This interaction stimulates cell cycle and proliferation-related proteins, thus enhancing hepatocyte proliferation and aiding the repair of liver damage (Nyström, 2021; Tsuchida and Friedman, 2017b). Moreover, the increased release of ECM components facilitates hepatocyte migration to injury sites, further contributing to liver repair (Nyström, 2021; Xia et al., 2020). However, chronic liver damage promotes the continuous activation of HSCs, their transformation into myofibroblasts, and the excessive secretion of ECM components (Gandhi, 2017; Tsuchida and Friedman, 2017b; Yang et al., 2019a; Zhang et al., 2021a). In contrast to chronic liver damage, after the initial liver damage, over-released ECM components are degraded by MMPs, allowing the re-establishment of liver architecture (Duarte et al., 2015). Therefore, the balance between ECM components synthesis and degradation is important for the healthy liver to recover under temporary liver damage. However, the activation of HSCs caused by chronic liver damage increases the activity of tissue TIMP-1 and TIMP-2, impeding MMPs function and leading to ECM components accumulation (Lachowski et al., 2019; Thiele et al., 2017; Duarte et al., 2015). This indicates that chronic liver damage can cause increased ECM synthesis and reduced ECM degradation, which ultimately breaks the balance of ECM remodeling.

During the progression of liver fibrosis, continuous accumulation of ECM components significantly alters the liver structure. Under physiological conditions, the components of ECM in a healthy liver maintain a certain balance to sustain the structure and function of liver (Duarte et al., 2015). In contrast, during chronic liver damage, the balance of ECM components is disrupted with the accumulation of ECM components such as collagen (Duarte et al., 2015; Villesen I et al., 2020). This augmented ECM deposition not only enhances hepatic stiffness, but also affects the blood supply and oxygen exchange in the liver. Specifically, the proliferation of fibrotic tissue results in vascular compression within the liver, thereby obstructing blood circulation. Reduced blood flow consequently impairs the oxygen delivery to the liver tissue, thereby undermining hepatocyte functionality (Wells, 2013; Peeters et al., 2018). Concurrently, emerging research has shown that the resultant hypoxic environment further instigates HSCs from a quiescent to an activated state (Cai et al., 2021; Trivedi et al., 2021; Khomich et al., 2019). These activated HSCs can produce more ECM components that aggravate cirrhosis and reduce oxygen delivery to liver tissue (Tsuchida and Friedman, 2017b; Duarte et al., 2015), creating a vicious cycle. Additionally, these activated HSCs exert significant influence on adjacent cells, including hepatocytes and immune cells, through the secretion of a spectrum of cytokines and chemokines (Tan et al., 2018; Gupta et al., 2019; Yang et al., 2019a). These neighboring cells can in turn affect more quiescent HSCs, indicating a secondary vicious cycle in the progression of liver fibrosis.

Therefore, a comprehensive understanding of ECM remodeling mechanisms is essential for the development of innovative therapeutic strategies for liver fibrosis. Future research should focus on modulating MMPs and TIMP activities to restore the ECM synthesis-degradation balance, potentially offering novel therapeutic strategies.

### 2.5 Molecular pathways and signaling mechanisms

**Fig. 2:**
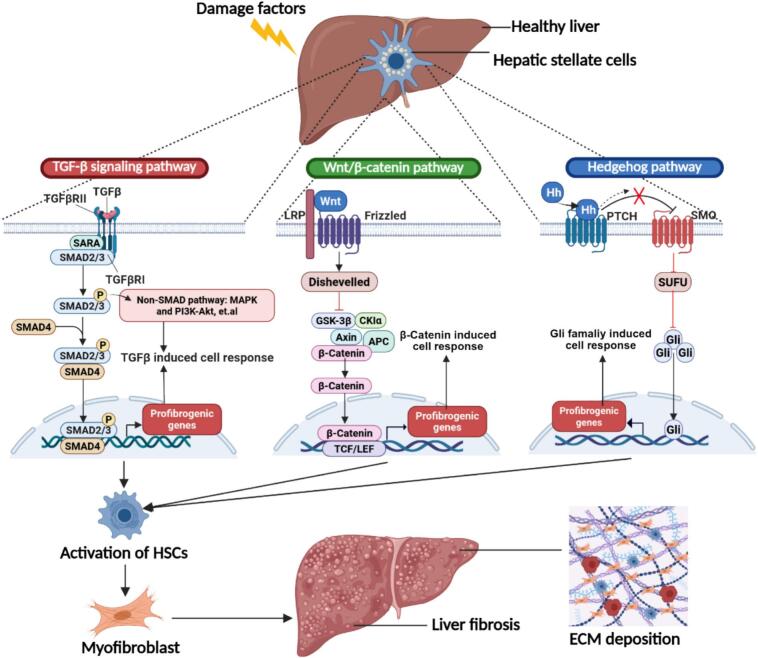
Key signaling pathways involved in HSCs activation and liver fibrosis. TGF-β, Wnt/β-catenin, and Hedgehog pathways promote the activation of immune cells and hepatic stellate cells (HSCs), leading to ECM accumulation and tissue sclerosis in the liver microenvironment.

Liver fibrosis is a complex biological process regulated by multiple cellular signaling pathways (Fig. 2). These pathways facilitate the activation of specific cell types, including immune cells and HSCs, which subsequently synthesize and deposit excessive ECM components, culminating in tissue sclerosis. Among them, TGF-β, Wnt/β-catenin and Hedgehog (Hh) are the most critical signaling pathways in liver fibrosis progression (Fabregat et al., 2016; Perugorria et al., 2019; Chung I et al., 2016a; Györfi et al., 2018).

#### 2.5.1 TGF-β signaling pathway

In a healthy liver, TGF-β mainly exists as an inactive large latent complex (LLC), which consists of TGF-β, latent TGF-β binding protein (LTBP), and latency-associated peptide (LAP) (Robertson I et al., 2015). Upon liver injury, the resulting inflammatory microenvironment activates immune cells to release cytokines, including TNF-α and interleukins (Robertson I et al., 2015; Robertson I and Rifkin, 2016; Huang et al., 2020). These mediators further stimulate HSCs and hepatic endothelial cells to secrete MMPs and Thrombospondin-1 (TSP-1). Subsequently, MMPs and TSP-1 act directly on LLCs in the ECM, resulting in the breaking of the junction between the TGF-β and LTBP and LAP, ultimately leading to the release of active TGF-β (Fabregat et al., 2016; Li et al., 2022b). These studies confirmed that chronic liver damage induces the release of active TGF-β in the liver microenvironment.

TGF-β drives ECM remodeling, which is essential for liver homeostasis, by activating both Smad and non-Smad signaling pathways in HSCs and upregulating ECM gene expression (Hu et al., 2018; Yoshida et al., 2018; Aashaq et al., 2022). Active TGF-β initially binds to the TβRII, inducing its conformational change and activation (Györfi et al., 2018). Subsequently, activated TβRII phosphorylates TβRI, which then phosphorylates Smad2 and Smad3 within HSCs. Phosphorylated Smads translocate from the cytoplasm to the nucleus. Within the nucleus, phosphorylated Smads bind to Smad4 and form a complex that functions as a transcription factor, increasing the synthesis of ECM components (Fig. 2) (Fabregat et al., 2016; Hata and Chen, 2016; Aashaq et al., 2022). In addition, activated TGF-β can transmit signals through non-Smad signaling pathways, including mitogen-activated protein kinase (MAPK) pathway and phosphoinositide 3-kinase (PI3K)/Akt pathway, both influential in ECM gene expression in the HSCs (Zhang, 2017; Finnson et al., 2020). Activation of TGF-β receptors initiates a signaling cascade beginning with the phosphorylation of Src homology 2 domain containing (Shc) transforming protein. The phosphorylated Shc protein can bind to growth factor receptor binding protein 2 (Grb2) and activate Son of sevenless (Ostrem et al., 2013) (Zhang, 2017; Luo, 2017; Peng et al., 2022). The complex formed by Grb2 and Sos can promote the activation of Raf kinase (Lin et al., 2022a; Peng et al., 2022). Then, Raf kinase phosphorylates and activates MAPK/ERK-activated kinase (MEK), ultimately leading to ERK activation (Peng et al., 2022). Past studies have shown that the activation of ERK pathway can enhance the synthesis of ECM proteins and upregulate pro-inflammatory cytokines (e.g., IL-6, TNF-α). thereby contributing to ongoing liver damage and fibrosis.

Activated TGF-β receptors also interact with PI3K, leading to activation of PI3K. As a lipid kinase, PI3K can phosphorylate phosphatidylinositol-4,5-bisphosphate (PIP2) at the cell membrane to phosphatidylinositol-3,4,5-triphosphate (PIP3). Then, PIP3 recruits Akt to the membrane, facilitating its subsequent phosphorylation and activation (Fruman et al., 2017; Fabregat et al., 2016). Activated Akt plays a significant role in the development of liver fibrosis. Previous studies showed that activated Akt can reduce the apoptosis of HSCs by inhibiting Bcl-2 (Zhang et al., 2019; Kim et al., 2020). Thus, an increased number of activated HSCs within the liver microenvironment can exacerbate the lesion.

#### 2.5.2 Wnt/β-catenin pathway

The Wnt/β-catenin signaling cascade represents a pivotal mechanism in the progression of liver fibrosis. When Wnt/β-catenin signaling is not activated, intracellular β-catenin is phosphorylated and degraded by a complex containing Axin, adenomatous polyposis coli (APC), glycogen synthase kinase 3β (GSK-3β), and casein kinase 1 (CK1) (Kim et al., 2017; Huang et al., 2019). However, liver damage triggers an upregulation of Wnt protein secretion by hepatic and immune cells. Wnt protein can bind to Frizzled receptor and low-density lipoprotein receptor-associated protein 5/6 (LRP 5/6) on the cell surface forming a receptor complex (Macdonald and He, 2012). Subsequently, the receptor complex can recruit and bind to Axin in the cell membrane, thereby preventing Axin forming a complex with other proteins to degrade β-catenin (Hua et al., 2018; Lybrand et al., 2019). These results suggest that the activation of Wnt/β-catenin signaling pathway can inhibit the degradation of intracellular β-catenin, leading to the excessive accumulation of β-catenin.

Some studies showed that intracellular accumulated β-catenin can translocate from the cytoplasm to nucleus, where it binds to T-cell factor/lymphoid enhancer factor (TCF/LEF) transcription factors (Doumpas et al., 2019). This β-catenin-TCF/LEF complex plays a critical role in upregulating the expression of genes associated with ECM production, thereby exacerbating liver fibrosis (Baarsma et al., 2011; Gao et al., 2023). Moreover, β-catenin induces immune cells to release a plethora of pro-inflammatory cytokines and promote the activation of quiescent HSCs, further accelerating the development of liver fibrosis (Li et al., 2023c).

In summary, the dysregulated activation of the Wnt/β-catenin signaling pathway is predominantly regarded as a detrimental factor in the pathogenesis of liver fibrosis. This aberrant activation contributes to excessive ECM deposition, culminating in the impairment of hepatic function.

#### 2.5.3 Hedgehog pathway

Hh ligands represent a crucial category of signaling molecules, pivotal in guiding the development of various organisms. Among them, sonic hedgehog (Shh) is a principal factor in the progression of liver fibrosis (Chung I et al., 2016b). Chronic liver damage can cause immune cells and hepatocytes to secrete some pro-inflammatory cytokines (Hammerich and Tacke, 2023b; Dixon et al., 2013). These cytokines can further increase the expression of Shh by activating some transcription factors (such as NF-κB) (Wang et al., 2016b).

Patch1 is a multi-channel transmembrane protein capable of receiving extracellular signals (Chung I et al., 2016a). In the absence of Hh ligands, Patch1 inhibits the activity of smoothened (Smok-Kalwat et al., 2023), suppressing the Shh signaling cascade. However, the engagement of Shh with Patch1 can negate this inhibition, thereby enabling the liberation and subsequent activation of SMO (Chung I et al., 2016a; Machado and Diehl, 2018). This process suggests that liver damage may activate SMO by inducing over-expression of Shh. The activation of SMO consequently triggers the activation of Glioma-associated oncogene (Gli) transcription factors within the nucleus (Machado and Diehl, 2018; Gao et al., 2018). In the context of liver fibrosis, the activation of Gli transcription factors assumes a critical role, orchestrating a range of pathological alterations. Activated Gli promotes the transition of quiescent HSCs into their activated form, leading to excessive production of ECM components and pro-inflammatory cytokines (Xie et al., 2013; Yan et al., 2020; Baghaei et al., 2022). Additionally, Gli upregulates ECM proteins such as collagen I/III and fibronectin, contributing to liver tissue remodeling, increased stiffness, and impaired hepatic function. Overall, the activation of the Hedgehog signaling pathway (Hh pathway) by Shh contributes to the progression of liver fibrosis through the stimulation of HSCs and the over-deposition of ECM components.

## 3 Drug delivery approach for liver fibrosis treatment

Liver fibrosis treatment should adopt a multi-targeted strategy that addresses critical stages of disease progression, including treatment of the primary disease or removal of causative factors, elimination of liver inflammation, inhibition of collagen fiber formation, promotion of apoptosis or transformation of activated HSCs back to a quiescent state, and direct promotion of the degradation of fibrous tissues.

### 3.1 Inhibiting hepatic stellate cells activation

HSCs make up about 10% of all resident liver cells and are present in the subendothelial space of Disse. In normal liver tissue, HSCs remain quiescent and non-proliferative. Quiescent hepatic stellate cells (qHSCs) are reservoirs of VA lipid droplets. Following liver injury, qHSCs transdifferentiate into activated hepatic stellate cells (aHSCs). aHSCs lose lipid droplet storage capacity and upregulate the expression of α-SMA, secreting a variety of signaling molecules to promote fibrosis progression (Tsuchida and Friedman, 2017a). A range of cytokines, including TGF-β, PDGF, and angiotensin II, promotes HSCs activation (Yan et al., 2021). Thus, blocking the activation of HSCs may be the most direct therapeutic target for the treatment of liver fibrosis.

TGF-β, as the most potent fibrotic cytokine, is secreted by various hepatic cells and drives fibrogenesis by activating HSCs. Thus, down-regulation of TGF-β expression or blockade of TGF-β pathway is effective in the treatment of liver fibrosis. Current research aims to enhance therapeutic efficacy through innovations in delivery technologies and multi-mechanism synergistic strategies. For example, a trivalent cholesterol (Chol)-conjugated DNA tetrahedral carrier (Chol-TD) leverages lipoprotein corona formation to improve hepatocyte uptake, delivering TGF-β1 antisense oligonucleotides to suppress mRNA and protein expression (Kim et al., 2022). Li et al. (Li et al., 2023a) synthesized an antagonistic peptide with anti-fibrillary effect that can self-assemble into nanoparticles. The nanoparticles selectively accumulated in the liver and had good antifibrotic activity by inhibiting the expression of TGF-β1. However, liver fibrosis involves multiple synergistic factors (e.g., ROS and hypoxic microenvironments), limiting the efficacy of TGF-β monotherapy. To address this, a ROS-clearing mesoporous polydopamine carrier (mPDA) was prepared with a porous structure that increased the drug loading of oxymatrine (OMT) and achieved liver homology targeting by coating the mPDA with an M2 macrophage exosome membrane. Among them, OMT can down-regulate the TGF-β/SMADS pathway and synergistically treat liver fibrosis with mPDA that can clear ROS (Tang et al., 2023). In addition, hypoxic liver further upregulates hypoxia-inducible factor-1α (HIF-1α) and aggravates hepatic hypoxia, thereby promoting HSC activation and leading to liver fibrosis(Baumann et al., 2016). Therefore, alleviating the hypoxia of liver tissue is an effective target for the treatment of liver fibrosis. Liu et al. (Liu et al., 2023a) developed nitrogen carbide nanosheets modified with VA to co-deliver graphene quantum dots (GQD) and small interfering RNA targeting HIF-1α (HIF-1α-siRNA). Polyethylene glycol (PEG) was introduced to improve the colloidal stability and blood circulation time of the nanosystem, thereby enhancing its *in vivo* delivery efficiency. The resulting nanosystem (VA-PEG-CN@GQD) exhibited effective liver-targeting ability. Under near-infrared light irradiation, GQD generate a large amount of oxygen to mitigate hypoxia, while HIF-1α-siRNA suppresses HSC activation by inhibiting the TGF-β1/Smad pathway, significantly improving liver fibrosis.

Although catalase (CAT), a biological macromolecule, effectively breaks down H_2_O_2_ (a form of ROS) into oxygen, its delivery to the liver remains challenging. Therefore, researchers encapsulated the anti-fibrotic drug saikosaponin b1 (Ssb1) in poly (lactic-*co*-glycolic acid) (PLGA) nanoparticles, constructing an MnO_2_@PLGA/Ssb1 nano-system [116]. This system not only decomposes H_2_O_2_ in a dose-dependent manner to produce oxygen but also downregulates HIF-1α expression, optimizing the fibrotic microenvironment of hypoxia. Together, these approaches provide a dual-regulated nanodrug delivery strategy for liver fibrosis treatment.

PDGF is one of the most important mitogens in the liver and plays a crucial role in the activation of HSCs. PDGF-bb binds to its receptor platelet-derived growth factor receptor-β (PDGFR-β), leading to the phosphorylation of tyrosine residues and initiating the MAPK signaling pathway, thereby stimulating the proliferation and activation of HSCs (Wang et al., 2016a). To inhibit this pathway, a nanopolyplex was designed to efficiently deliver negatively charged PDGFR-β siRNA (siPDGFR-β). This nanopolyplex consists of a cationic Poly (aspartic acid) block complex and a ROS-responsive dithioketal crosslinker that prevented the premature release of siPDGFR-β from the nanopolyplex core (T-C-siPDGFR-β). Due to its surface modification with VA, it can hijack retinol-binding protein (RBP) in the blood to form a protein corona, enhancing its stability in blood circulation and facilitating targeted delivery to HSCs *via* retinol-binding protein receptor (RBPR), which is overexpressed in HSCs. When the concentration of siPDGFR-β exceeds 40 nM, PDGFR-β mRNA expression is minimized, effectively blocking the PDGF-BB/PDGFR signaling axis responsible for HSC activation (Huang et al., 2023). However, VA-modified nanopolymers may also be hijacked by hepatocytes to reduce their targeting effectiveness toward HSC. Li et al. (Li et al., 2023d) synthesized a liposome with a CREKA-targeting peptide with the antifibrotic drug sorafenib (SOR) loaded (CREKA-Lip/SOR). CREKA-targeting peptides can actively target fibronectin, which is highly expressed in aHSCs, enabling selective delivery of SOR. CREKA-Lip/Sor significantly enhances the distribution of SOR in the liver while decreasing the distribution of SOR in the heart. SOR accumulating in the liver prevents PDGF-induced HSCs activation and proliferation by inhibiting phosphorylation of PDGFR-β.

Previous studies have shown that low molecular weight fibroblast growth factor 2 (FGF2) possesses notable antifibrotic properties (Pan et al., 2015). FGF2 exerts its effect by binding to fibroblast growth factor receptor 1 (FGFR1), which is highly expressed by HSCs, and inhibiting the activation of HSCs. However, FGF2 suffers from enzymatic degradation and poor stability. To overcome the limitations of conventional antifibrotic agents, engineered superparamagnetic iron oxide nanoparticles (SPIONs) functionalized with endothelin A receptor antagonists were developed to specifically target aHSCs. By inhibiting the endothelin-1/endothelin A receptor (ET-1/ETAR) signaling pathway, the nanosystem effectively suppressed HSC activation and alleviated liver fibrosis (Hove et al., 2024).

aHSCs significantly upregulate the expression of C-X-C motif chemokine receptor 4 (CXCR4) to bind more stromal cell-derived factor-1 (SDF-1) to further stimulate HSCs activation (Chen et al., 2014). In order to successfully deliver the CXCR4 antagonist AMD 3100 (plerixafor) to HSCs and achieve the successful release of small molecule drugs, a ROS-responsive nanoparticle was developed using natural lipoic acid, which responds to elevated ROS levels in the liver. These nanoparticles can co-deliver AMD 3100 and SOR to achieve synergistic therapeutic effects on liver fibrosis (Sun et al., 2024). ROS-responsive drug delivery systems have shown considerable potential for liver fibrosis therapy due to the elevated oxidative stress microenvironment in fibrotic liver tissue. Bilirubin-based nanoplatforms, particularly chitosan-bilirubin self-assembled micelles encapsulating losartan, can undergo ROS-triggered structural disruption and achieve controlled drug release in fibrotic regions. These ROS-responsive nanosystems effectively suppress HSC activation and reduce collagen deposition and α-SMA expression, thereby alleviating the progression of liver fibrosis (Liu et al., 2024).

Some naturally active compounds extracted from plants, such as forsythiaside A (FA), chlorogenic acid (CGA), and resveratrol (RES), exhibit the ability to suppress HSC activation. However, their clinical applications remain limited by poor stability and inefficient delivery. Recent studies have demonstrated that hyaluronic acid (HA)-modified antioxidative nanomedicines can specifically target CD44-overexpressing activated HSCs and effectively alleviate liver fibrosis by regulating oxidative stress and fibrotic signaling pathways. These biomimetic nanoplatforms significantly improve cellular uptake and antifibrotic efficacy compared with free drugs (Shinn et al., 2024). Due to its hydrophilic nature, CGA exhibits poor membrane permeability. As an emerging 3D nucleic acid nanomaterial, tetrahedral framework nucleic acid (tFNA) is a potential drug delivery system. For the purpose of improving the membrane penetration efficiency of CGA and reduce its dose, CGA was loaded into tFNAs to achieve its efficient delivery (tFNAs-CGA). tFNAs-CGA can significantly enhance the uptake of CGA in LX2 and reduce the concentration of CGA *in vivo* (Yao et al., 2023). To increase the solubility and prolong the biological half-life of RES, it was loaded into ROS-responsive micelles. Poly (L-methionine-*block*-*N*-trifluoroacetyl-l-lysine) (PMK) conjugated with a cyclic arginine-glycine-aspartic acid (CRGD) peptide, which then self-assembled into micelles and encapsulated RES (CRGD-PMK-MCs). The CRGD peptide targets VI collagen, which is highly expressed in aHSCs. Under high intracellular ROS conditions, the micelles can realize the conversion from hydrophobicity to hydrophilicity, triggering the release of RES. The results showed that CRGD-PMK-MCs reduced ROS levels in liver tissues and inhibited the activation of HSCs by down-regulating TGF-β and collagen expression (Hao et al., 2022). Due to the unique physiology of the liver, nanoparticles must traverse liver sinusoidal endothelial cells (LSECs), which contain fenestrae with an average diameter of approximately 100 nm, forming a natural barrier to drug delivery targeting aHSCs. Therefore, careful control of nanoparticle size is essential for efficient translocation across LSECs. Luo et al. used the precipitation method of acid-base neutralization reaction to prepare silibinin (SLB) nanocrystals with a drug loading of up to 49.4%. This nanocrystal adsorbs human serum albumin (HSA) with a particle size of about 60 nm on their surface. The HSA enables binding to cysteine-rich acidic secreted protein (SPARC), which is highly expressed on HSCs, through the Disse space, thereby promoting endocytosis and enrichment in fibrotic liver. SLB-HSA NC has good anti-fibrotic ability and is an effective means for the treatment for liver fibrosis (Luo et al., 2023).

### 3.2 Reprogramming of aHSCs into qHSCs

Liver fibrosis is a chronic reversible disease that can be reversed when the source of chronic injury is removed (Kisseleva and Brenner, 2021b). Given the significant plasticity of HSCs, aHSCs also have the potential to differentiate into qHSCs.

The Hh pathway is an important regulator of liver fibrosis. SMO is a component of the Hh pathway, and down-regulation of SMO expression has been shown to inhibit the activation of HSCs (Kim et al., 2021). Inhibition of both the Hh pathway and the TGF-β pathway can reprogram aHSCs into qHSCs, thereby restoring normal liver function. Younis et al. (Younis et al., 2023) constructed lipid nanoparticles (LNPs) with CL15H6 as a predominantly lipid material, which can efficiently deliver SiSMO and SiTGF-β to aHSC. CL15H6 is an ionizable lipid that can achieve 70% knockout efficiency at a low dose of 0.25 mg/kg. However, unmodified LNPs can only accumulate in liver tissue *via* passive targeting. To increase the accumulation of drugs in liver tissue, it is necessary to design a drug delivery vehicle that actively targets liver tissue. Extracellular vesicles (EVs, 40–160 nm) are important mediators of intercellular communication and have emerged as promising cell-free therapeutic platforms due to their low immunogenicity, intrinsic biocompatibility, and modifiable surface properties. Recent advances in engineered EV-based delivery systems have highlighted their potential for targeted therapy in fibrotic and inflammatory diseases (El Andaloussi et al., 2013). Studies have shown that mesenchymal stem cell (MSC)-derived EVs possess therapeutic potential in inflammatory diseases. In order to enhance the specific targeting ability of MSC-EVs toward aHSCs, hydrophobic VA was inserted into MSC-EVs to target aHSCs and improve the anti-liver fibrosis ability of MSC-EVs. *In vitro*, the fluorescence intensity of V-EV in aHSCs was 1.8 times higher than that of EVs. *In vivo* biodistribution, V-EV highly overlapped with aHSCs fluorescence signals. Both *in vitro* and *in vivo* results confirmed that V-EV had a strong targeting ability toward aHSCs (You et al., 2021). HSCs resemble cancer cells in that they activate and proliferate themselves by upregulating the glycolytic pathway (Mejias et al., 2020). Therefore, inhibition of the HSCs glycolytic pathway may be a potential strategy for liver fibrosis treatment. Researchers have developed VA-modified HSC-targeting micelles loaded with camptothecin to suppress HIF-1α and its downstream glycolytic pathway. The micelles can remain in the liver for up to 48 h, laying the foundation for the liver fibrosis treatment. Meanwhile, the micelles can reverse aHSCs to a quiescent state by inhibiting glycolysis of HSCs to achieve an effective therapy of liver fibrosis (Xiang et al., 2023).

### 3.3 Degradation of fibrotic extracellular matrix

Liver fibrosis is caused by excessive deposition of the ECM due to chronic inflammation (Zuo et al., 2023). The dense ECM causes hepatic sinusoidal nerves to constrict, interferes with normal blood flow, and impairs the structure and function of the liver (Luangmonkong et al., 2023). Moreover, ECM also hinders nanoparticles from reaching fibrotic sites, resulting in reduced therapeutic efficacy. Thereby, focusing on restoring the normal function of the ECM represents a promising strategy for the treatment of liver fibrosis.

Collagen (mainly collagen-I), the major component of the fibrous liver ECM, is significantly upregulated in fibrotic liver tissue. MMP-1, a key enzyme in the ECM responsible for the degradation of collagen-I, is typically downregulated in fibrosis conditions. To address this, E. Geervliet et al. (Geervliet et al., 2021) developed a polymeric vesicle (Psome) capable of encapsulating MMP-1 while maintaining its enzymatic activity, promoting the degradation of fibrotic ECM and thereby alleviating liver fibrosis.

**Fig. 3:**
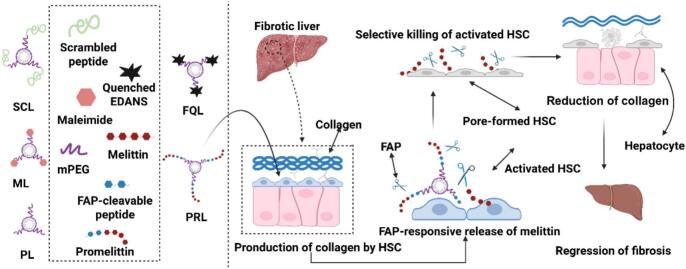
Inhibition of collagen production *via* targeted melittin delivery. Melittin-functionalized liposomes release melittin in FAP-overexpressing environments, selectively disrupting activated hepatic stellate cells (aHSCs) and reducing collagen overproduction in the fibrotic ECM.

In addition to accelerating collagen degradation, inhibition of collagen production can alleviate fibrotic ECM at the source. Collagen production can be indirectly inhibited by reducing aHSCs. Fibroblast activation protein (FAP) is specifically expressed in aHSCs, and the preparation of FAP-responsive nanocarriers is an effective method to target liver fibrosis. Studies have shown that, melittin binds to the surface of functionalized liposomes (Fig. 3) and is released in environment with high FAP expression. The released melittin specifically disrupts the cell membrane of aHSCs, depleting their population and thereby suppressing collagen fiber overproduction (Lee et al., 2022). Additionally, heat shock protein 47 (HSP47) is a collagen-specific chaperone, and downregulation of HSP47 expression effectively reduces collagen deposition. Han et al. (Han et al., 2023) synthesized a SiHSP47-loaded LNPs using microfluidic hybrid technology. The LNPs, functionalized with benzamide-based lipids (AA-T3A-C12), selectively target σ receptor-overexpressing HSCs in the liver, resulting in approximately 65% silencing of HSP47. Knockdown of HSP47 successfully decreased collagen deposition and alleviated liver fibrosis.

In the ECM, the balance between collagen synthesis and degradation is regulated by MMPs and tissue inhibitors of metalloproteinases (TIMPs) (Shan et al., 2023). Given that MMP/TIMP imbalance results in excessive collagen deposition, a promising approach to treating liver fibrosis is a dual-targeting strategy that simultaneously inhibits collagen production and promotes its degradation. Zn^2+^ has a hepatoprotective effect by inhibiting the activity of proline-4 hydroxylase (P4H) which promotes collagen production. Liang et al. (Liang et al., 2023). constructed a biomimetic nanomodulator coated by platelet membrane, which can be loaded with TIMP-1 circular DNAzyme (cirDNAzyme) through zeolitic imidazolate framework-8 (ZIF-8). ZIF-8 can release Zn^2+^ and cirDNAzyme in lysosomes, Zn^2+^ suppresses P4H activity, while cirDNAzyme downregulates TIMP-1 mRNA levels by 33.8%, synergistically reducing collagen accumulation and enhancing degradation, effectively alleviating fibrosis. Another study employed LNPs to deliver siCol1α1 and siTIMP-1, and the lipid Chol-PEG-VA could be specifically targeted to HSCs. siCol1α1 inhibits collagen synthesis, whereas siTIMP-1 promotes degradation, achieving a dual therapeutic effect against fibrosis (Qiao et al., 2020). By inhibiting the activation of HSCs, collagen production can also be inhibited. Gu et al. prepared a silica cross-linked micelle that could load rosiglitazone on its hydrophobic core, modifying FEN 1 and hpDNA probes onto the end groups of surface PEG ligands for ribonucleoprotein (RNP) delivery. When micelles entered cells through CTCE 9908 targeting aHSCs-overexpressed CXCRs 4, RNPs could suppress the expression of TIMP-1 to promote collagen degradation, and rosiglitazone could inhibit the activation of HSCs to reduce collagen production. The micelles can reduce the fibrosis area of carbon tetrachloride (CCl_4_)-induced liver fibrosis mice to 1.3 ^±^ 0.2%, which is close to healthy liver (Gu et al., 2023). Physical methods (Sonoporation) to increase the permeability of nanoparticles in fibrotic ECM is an emerging method. Chen et al. (Chen et al., 2023) prepared a microbubble loaded with hydroxycamptothecin (HCPT) (HCPT@MBs) to assist it in passing through the delivery barrier by acoustic porosity. The experimental results showed that compared with free HCPT and HCPT-LIP, HCPT@MBs could deliver HCPT to fibrotic liver more effectively.

Besides sonoporation, additional strategies for overcoming ECM-mediated transport barriers warrant further discussion. Excessive ECM deposition, particularly collagen accumulation, forms a dense fibrotic network that restricts nanoparticle diffusion and limits drug accessibility to aHSCs within fibrotic lesions (Zhao et al., 2023; Acharya et al., 2021). To address this physical barrier, enzyme-mediated ECM remodeling has emerged as a promising strategy to facilitate drug penetration. For instance, collagenase I-modified nanocarriers have been developed as nanodrills that actively degrade the surrounding type I collagen barrier, thereby reducing steric hindrance and improving the intratissue delivery efficiency of therapeutic agents in fibrotic tissue (Fan et al., 2020). In addition, studies have shown that fibrosis progression is often accompanied by an imbalance between MMPs (such as MMP-2 and MMP-9) and their tissue inhibitors (TIMPs), resulting in impaired endogenous ECM degradation. Therefore, the delivery of exogenous enzymes to restore matrix remodeling has emerged as a key therapeutic intervention (Ortiz et al., 2021). However, uncontrolled enzymatic activity may disrupt hepatic microarchitecture, highlighting the need for targeted and stimuli-responsive matrix remodeling systems to ensure both efficacy and safety (Kisseleva and Brenner, 2021a).

Another strategy focuses on matrix softening rather than direct degradation. Liver fibrosis is characterized by increased matrix stiffness, which contributes to HSC activation and impaired tissue permeability (Kostallari et al., 2022). Therefore, suppressing collagen crosslinking or reducing ECM rigidity may improve nanoparticle penetration into fibrotic tissue (Klepfish et al., 2020). In addition, reprogramming aHSCs into qHSCs may indirectly attenuate ECM accumulation and stiffness, creating a more permissive microenvironment for drug delivery (Schwabe and Brenner, 2025).

Furthermore, fibrosis microenvironment-responsive nanocarriers have emerged as a promising strategy to improve therapeutic delivery in fibrotic liver tissue. Liver fibrosis is characterized by pathological microenvironmental alterations, including excessive ECM deposition, elevated ROS, hypoxia, and dysregulated tissue remodeling, which may be exploited to achieve site-specific drug release and enhanced therapeutic selectivity (Zhang et al., 2024b). In particular, ROS-responsive nanoplatforms have demonstrated considerable potential in fibrosis-targeted therapy. For example, Surendran et al. developed a bilirubin-conjugated chitosan nanotheranostic system that responds to elevated ROS levels in fibrotic tissue, enabling ROS-triggered structural destabilization and controlled drug release at pathological sites, thereby enhancing localized therapeutic action in liver fibrosis (Surendran et al., 2020). These findings suggest that nanocarriers capable of responding to fibrosis-associated pathological cues may represent a promising strategy for improving therapeutic precision while minimizing systemic adverse effects (Zhao et al., 2026).

### 3.4 Inhibiting hepatic inflammation

As one of the pathogenic factors of liver fibrosis, inflammation plays a leading role in the process of liver fibrosis through the interplay of inflammatory cells, cytokines, and inflammation-related signaling pathways (Han-Jing Zhangdi et al., 2019). Persistence of inflammation causes normal liver tissue to be replaced by fibrotic scar tissue, resulting in structural and dysfunctional liver (Hammerich and Tacke, 2023a).

Macrophages play a pivotal role in liver fibrosis by recruiting to inflamed tissue, secreting pro-inflammatory cytokines, and regulating ECM deposition (Ramachandran et al., 2019). The macrophage phenotype is divided into pro-inflammatory M1 and anti-inflammatory M2. Therefore, reversing the M1 macrophage phenotype to the M2 macrophage phenotype may serve as an effective strategy for liver fibrosis. A study developed LNPs (MUA/Y) co-loaded with small-molecule compound AC37 (AC37) and small interfering RNA targeting ubiquitin-specific protease 1 (siUSP1), surface-modified with milk fat globule-EGF factor 8 (MFG-E8) protein. MFG-E8 promotes the repolarization of pro-inflammatory M1 macrophages toward an anti-inflammatory M2 phenotype to resolve inflammation. Additionally, MFG-E8 enhances macrophage phagocytosis of collagen. These actions collectively contribute to increased drug accumulation in HSCs. AC37 can inhibit the activation and proliferation of HSCs in synergy with SiUSP1. Immunofluorescence staining showed that MUA/Y significantly reduced inducible nitric oxide synthase (iNOS) (an M1 marker) while increasing CD206 (an M2 marker), demonstrating its ability to modulate macrophage phenotypes, providing a new treatment option for the treatment of liver fibrosis (Duan et al., 2023). C-C Chemokine Receptor 2 (CCR2) is highly expressed in fibrotic livers, which can lead to liver fibrosis by recruiting M1 macrophages and activating HSCs (Kaps et al., 2022). Inhibiting CCR2 may alleviate fibrosis, a tetrahedral framework DNA nanostructure (tFNA) was prepared that could be loaded with siCcr2 to knock down the expression of CCR2 (tFNAsiCcr2). tFNAsiCcr2 mainly inhibited the hepatic accumulation of inflammatory fascin actin-bundling protein 1-positive (FSCN1^+^) macrophages and HECT and RLD domain containing E3 ubiquitin protein ligase family member 6-positive (HERC6^+^) neutrophils, ameliorating liver fibrosis by inhibiting inflammation and constructing an anti-fibrotic microenvironment (Tian et al., 2023). HMGB1 promotes collagen deposition by activating the PI3K-Akt pathway and stimulates macrophages to release pro-inflammatory cytokines, accelerating fibrosis (Zhou et al., 2022a). Zhang et al. (Zhang et al., 2020) developed a lipid nanoparticle modified with the pPB peptide and loaded with HMGB1 siRNA (HMGB 1 siRNA @SNALP-pPB), which downregulated HMGB1 expression and suppressed inflammation and fibrotic progression.

Liver microbiota dysbiosis has emerged as a key pathogenic factor in liver fibrosis. Cyclodextrin (Macdonald and He, 2012) has been shown to have prebiotic properties and restore bacterial homeostasis (Ren et al., 2022). By conjugating PEG 2000 with VA and CD to form VAP2000@CD, followed by self-assembly with the anti-inflammatory compound dihydrodanshensu I (DHI), researchers developed a nanoparticle (DHI-VAP2000@CD) that effectively inhibits the expression of inflammatory cytokines. DHI-VAP2000@CD can greatly increase the ratio of M1/M2 macrophages and reduce pro-inflammatory cytokines release, and restore liver microbial balance, thereby mitigating liver fibrosis.

### 3.5 Blocking the undesirable crosstalk between HSCs and KCs

As mentioned earlier, aHSCs play a central role in the process of liver fibrosis. The location of aHSCs within the space of Disse presents significant challenges for drug delivery systems to effectively target. Notably, KCs make up about 20% of the non-parenchymal liver cells, in contrast to HSCs, which account for about 5% (Li et al., 2022a). Moreover, because KCs are located within the hepatic sinusoids, they can come into directly contact with various blood components, including drug delivery systems. On the one hand, in the early stage of liver fibrosis, liver parenchymal cell death stimulates KCs to produce ROS, cytokines, chemokines and pro-inflammatory factors, which directly stimulate HSCs activation and then lead to exacerbate liver fibrosis. On the other hand, PDGF secreted by KCs can induce aHSCs migration. Given the interaction between KCs and aHSCs, blocking the crosstalk between them may be another strategy in the treatment of liver fibrosis.

Qiu et al.(Qiu et al., 2023) constructed an ultra-small nanoparticle (*CF*) composed of a Cu-Fe bimetal, which was then encapsulated *CF*@BPPM to form a hybrid polymer composed of a baicalin prodrug (BP) and a mannose derivative (Liu et al.), forming *CF*@BPPM. Mannose can specifically bind to the mannose receptor with high expression of KCs, thereby increasing the uptake of *CF*@BPPM by KCs. Within the KCs lysosomes, the mixed polymer was acidified and deconstructed, releasing Cu^2+^, Fe^2+^ and baicalin. The released Cu^2+^ and Fe^2+^ can inhibit the secretion of PDGF by upregulating the expression of heme oxygenase (HO-1). In addition, baicalein can inhibit the secretion of TGF-β to inhibit the activation of HSCs. *CF*@BPPM can improve liver fibrosis by directly acting on KCs and inhibiting KCs from secreting various inflammatory factors and cytokines, thereby blocking the crosstalk between KCs and HSCs (Qiu et al., 2023). A polymer loaded with both CXCR4 antagonist and anti-miR155 was prepared. The polymer targets the liver *via* mannose, and its loaded CXCR4 antagonist can revert aHSCs to qHSCs, and anti-miR155 knocks down high-expressed miR155 in KCs to alleviate liver inflammation.

### 3.6 Promoting autophagy in HSCs or macrophages

Autophagy is a lysosome-dependent intracellular degradation process that maintains cellular homeostasis by eliminating damaged organelles, misfolded proteins, and protein aggregates (Zhao et al., 2025a; Kundu et al., 2025). During the development and progression of liver fibrosis, autophagy exhibits pronounced cell type-specific functions. In aHSCs, autophagy promotes the degradation of VA-containing lipid droplets, providing energy and metabolic substrates for HSCs activation and ECM synthesis, thereby facilitating fibrogenesis (Liu et al., 2025a). Consequently, autophagy in aHSCs is generally considered a pro-fibrotic mechanism. In contrast, autophagy in macrophages promotes the clearance of profibrotic mediators, apoptotic cells, and cellular debris, thereby enhancing tissue repair and inflammation resolution and exerting anti-fibrotic effects (Liu et al., 2025a; Vitaliti et al., 2025). Therefore, selectively inhibiting autophagy in aHSCs while enhancing autophagy in macrophages has emerged as a promising strategy for precision anti-fibrotic therapy.

Accumulating evidence has demonstrated that genetic or pharmacological inhibition of autophagy in aHSCs can effectively attenuate liver fibrosis (Hou et al., 2024; Huang et al., 2025). Key autophagy-related regulators, including autophagy-related protein 5 (Atg5), autophagy-related protein 7 (Atg7), and p62, have been widely explored as therapeutic targets through siRNA-mediated silencing or pharmacological inhibition using small-molecule agents such as chloroquine and hydroxychloroquine (Hou et al., 2024; Le et al., 2022; Lin et al., 2024; Cheng et al., 2024; Zhang et al., 2021b). To improve therapeutic specificity and minimize systemic toxicity, various HSC-targeted delivery systems have been developed. For example, VA-modified liposomes exploit the high expression of retinol-binding protein receptors on aHSCs to achieve selective uptake (Lai et al., 2024). Similarly, mannose-6-phosphate (M6P)-modified nanoparticles can target the M6P/IGF-II receptor expressed on aHSCs, enabling the precise delivery of autophagy inhibitors (Beljaars et al., 2001; Ye et al., 2006). In a representative study, cyclic RGD peptide-modified liposomes exhibited enhanced accumulation in aHSCs and significantly reduced collagen deposition in a CCl_4_-induced mouse model of liver fibrosis (Chai et al., 2012).

Conversely, autophagy in macrophages generally exerts anti-fibrotic effects. It not only promotes the polarization of macrophages from a pro-inflammatory M1-like phenotype to a pro-reparative M2-like phenotype, but also enhances efferocytosis and suppresses NLRP3 inflammasome activation, thereby alleviating inflammation and facilitating tissue repair (Zhao et al., 2025b; Zhang et al., 2025b; Shi et al., 2025). Autophagy inducers such as rapamycin, trehalose, and spermidine have demonstrated significant anti-fibrotic efficacy in various experimental models of liver fibrosis (Kaushal et al., 2010; Liu et al., 2019; Tung et al., 2024). However, systemic activation of autophagy may simultaneously promote HSC activation and compromise therapeutic outcomes, highlighting the importance of macrophage-specific drug delivery. Currently, mannose-modified nanocarriers can target alternatively activated macrophages through recognition of the CD206 receptor, whereas folate-modified nanoparticles selectively bind folate receptor β, which is highly expressed on macrophages in fibrotic livers (Kaps et al., 2020; Kumar et al., 2026; Ibrahim I et al., 2023).

Recent advances in nanomedicine have enabled simultaneous or sequential modulation of autophagy in different cell populations. Stimuli-responsive nanocarriers can achieve controlled drug release in response to the unique characteristics of the fibrotic microenvironment. For example, ROS-responsive nanoparticles containing thioketal linkers undergo degradation and release their payloads in ROS-rich fibrotic liver tissues, where both aHSCs and inflammatory macrophages exhibit elevated ROS levels (Sun et al., 2024; Liu et al., 2025b). In addition, MMP-responsive gelatin-coated liposome has been developed to exploit the overexpression of MMPs within fibrotic lesions, allowing drug release in response to MMP-2 and MMP-9 (Togami et al., 2025).

Overall, the successful application of autophagy-targeted therapy for liver fibrosis depends on precise cell type-specific regulation. With the rapid development of ligand-modified nanocarriers and stimuli-responsive drug delivery systems, selective modulation of autophagy in HSCs and macrophages has shown considerable therapeutic potential. Future studies are needed to further validate the efficacy and safety of these strategies in clinically relevant models and facilitate their clinical translation.

### 3.7 Multiple mechanisms for the treatment of liver fibrosis

Liver fibrosis is caused by a variety of factors, so a combination of two or more treatment strategies is expected to achieve better treatment outcomes.

**Fig. 4:**
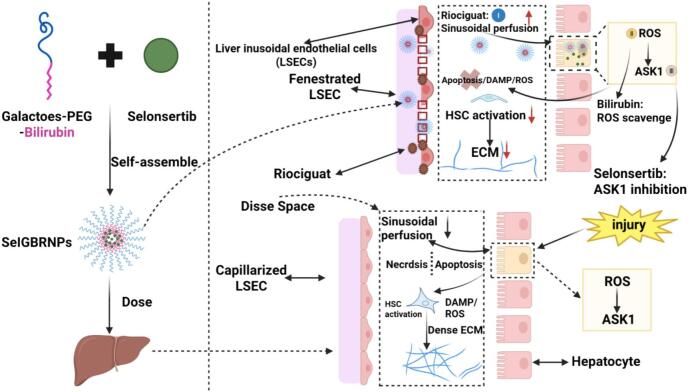
Hepatocytes-targeting micelles. Self-assembled micelles composed of β-D-galactose-polyethylene glycolated bilirubin (Gal-PEG-BR) are used to co-deliver riociguat and selonsertib. The β-D-galactose moiety enables specific targeting to hepatocytes.

Persistent liver damage leads to hepatocyte death and the release of intracellular contents. ROS released from damaged hepatocytes activates KCs and HSCs, triggers the initiation of the inflammatory cascade, and accelerates the progression of liver fibrosis. Therefore, the elimination of ROS will be combined with other fibrosis pathogenesis to provide a promising strategy for the treatment of liver fibrosis. Studies have shown that small-sized carbon quantum dots have ROS scavenging effects due to their structural defects and active groups, and can achieve effective enrichment in hepatocytes. Dexamethasone was loaded onto carbon dots (CDs) to treat liver fibrosis by eliminating hepatocyte ROS and inhibiting inflammation. Li et al. (Li et al., 2023b) prepared a micelle formed by self-assembly of β-D-galactose-polyethylene glycolated bilirubin polymer (Gal-PEG-BR) that can be used to co-deliver riociguat and selonsertib. β-D-galactose targets hepatocytes for specific delivery of therapeutics (Fig. 4). Bilirubin can clear ROS in liver tissue, riociguat can enhance sinus perfusion and reduce ROS production, and selonsertib reduces hepatocyte apoptosis by inhibiting apoptosis signal-regulating kinase 1 (ASK1). The polymer micelles can achieve dual regulation of enhanced sinusoidal perfusion and inhibition of hepatocyte apoptosis, effectively improving the liver function.

Although ligand-modified nanoparticles have improved liver-targeting efficiency, several targeting ligands still suffer from limited cellular specificity (Kaps et al., 2023; Yuan et al., 2024). For example, VA-mediated targeting may also induce nanoparticle uptake by hepatocytes due to physiological retinol metabolism in the liver. Similarly, mannose-modified systems are susceptible to nonspecific clearance by KCs and splenic macrophages because mannose receptors are broadly expressed in the mononuclear phagocyte system (Colino I et al., 2020).

HA-based targeting strategies may also exhibit off-target effects because CD44 is expressed in multiple inflammatory and immune cell populations. In addition, fibronectin-targeting peptides such as CREKA may accumulate in non-fibrotic inflammatory tissues where fibronectin is overexpressed (Chen et al., 2024).

Therefore, future studies should focus on improving targeting specificity through dual-ligand strategies, stimuli-responsive activation, or microenvironment-adaptive nanoplatforms to minimize off-target accumulation and enhance therapeutic precision (Yuan et al., 2024).

### 3.8 Comparative advantages and limitations of nanoparticle systems for liver fibrosis therapy

Although various nanoparticle-based delivery systems have demonstrated promising therapeutic potential in liver fibrosis, each platform possesses distinct advantages and limitations that should be critically considered (Table 1). Liposomes exhibit favorable biocompatibility, high drug-loading flexibility, and relatively mature manufacturing processes, making them attractive for clinical translation. However, their rapid uptake by KCs and limited penetration through dense fibrotic ECM may reduce therapeutic efficiency (Zhang et al., 2026b; Canestrale et al., 2025b). Polymeric micelles are particularly suitable for hydrophobic drug delivery and can be engineered to respond to pathological stimuli such as ROS. Nevertheless, concerns regarding premature drug leakage and insufficient *in vivo* stability remain (Yang et al., 2019a; Zhang et al., 2026b). EVs and exosome-based systems possess intrinsic biocompatibility, low immunogenicity, and natural intercellular communication capabilities, enabling efficient targeting of activated HSCs. Despite these advantages, challenges associated with large-scale production, purification, and batch-to-batch variability hinder their clinical application (Liu et al., 2023c; Hu et al., 2023). Inorganic nanoparticles offer multifunctional therapeutic potential, including ROS scavenging, oxygen generation, and imaging capabilities, but concerns regarding long-term biosafety, biodegradability, and accumulation in tissues remain unresolved (Liu et al., 2025c; Jin et al., 2020; Sun et al., 2021). Therefore, future development of antifibrotic nanomedicines should focus on balancing targeting efficiency, biocompatibility, scalability, and safety according to specific therapeutic objectives (Maria Giovanna Armillotta et al., 2025).

**Table 1:** Comparative advantages and limitations of representative nanoparticle systems for liver fibrosis therapy.

Nanoparticle system	Major advantages	Major limitations	Typical applications in liver fibrosis	References
Liposomes	Excellent biocompatibility, flexible drug-loading capability for hydrophilic and hydrophobic agents, relatively mature manufacturing process with translational potential	Rapid uptake by Kupffer cells may reduce liver targeting efficiency, limited penetration through dense fibrotic ECM	Delivery of antifibrotic drugs and nucleic acids, clinically translatable nanocarriers	(Zhang et al., 2026b, Canestrale et al., 2025a)
Polymeric micelles	Particularly suitable for hydrophobic drug delivery, tunable physicochemical properties, can be engineered as ROS-responsive or stimuli-responsive systems	Premature drug leakage, insufficient *in vivo* stability and circulation persistence	ROS-responsive delivery of antifibrotic agents such as resveratrol and losartan	(Yang et al., 2019a, Surendran et al., 2020, Hao et al., 2022)
Extracellular vesicles (EVs)/Exosomes	Intrinsic biocompatibility, low immunogenicity, natural intercellular communication capability, efficient targeting toward activated HSCs	Challenges in large-scale production, purification, storage, and batch-to-batch consistency	Cell-free delivery systems with intrinsic liver-targeting and signaling functions	(Liu et al., 2023b, Hu et al., 2023, Shen et al., 2023)
Inorganic nanoparticles	Multifunctionality, including ROS scavenging, oxygen generation, imaging, and theranostic applications, high structural stability	Concerns regarding long-term biosafety, biodegradability, tissue accumulation, and potential toxicity	Oxygen-generating systems, magnetic nanoparticles, imaging-guided antifibrotic therapy	(Liu et al., 2025c, Jin et al., 2020, Sun et al., 2021, Eftekhari et al., 2021)

### 3.9 Current status of clinical translation

While decades of basic research have largely elucidated the core mechanisms of liver fibrosis, the clinical translation of therapeutics remains fraught with formidable obstacles. To date, the number of drugs that have successfully completed clinical trials, gained market approval, and are indicated for liver fibrosis and its associated conditions is exceedingly limited. Table 2 summarizes representative conventional drugs and nanomedicines that have entered clinical development. As shown, although some progress has been made in treating these diseases, failures represent the majority, and a significant gap remains before ideal therapeutic regimens are realized.

**Table 2:** Approved drugs and nanomedicine-based therapeutics for liver fibrosis: current clinical development status.

Drug and nanodrug names	Approval year	Formulation type	Loaded drugs	Clinical applications	Target and mechanism	Development stage	Clinical translation bottleneck	Administration route	Agency	References
Resmetirom	2024	Small molecule	Resmetirom	NASH with moderate to advanced liver fibrosis	THR-β selective agonist	Approved	First FDA-approved NASH fibrosis drug, limited fibrosis reversal rate (25.9–29.9%), long-term safety data accumulating.	Oral	FDA	(Harrison et al., 2024, Brett, 2024)
Semaglutide (Wegovy)	2025	Small molecule (GLP-1 agonist)	Semaglutide	MASH with moderate-to-advanced fibrosis (F2–F3)	GLP-1 receptor agonist	Approved	Primary mechanism *via* weight loss and metabolic improvement, indirect anti-fibrotic effect.	Subcutaneous injection	FDA	(Jara et al., 2025)
ESBRIET®	2014/2011	Small molecules	Pirfenidone	IPF	TGF-β signaling pathway inhibitor, inhibition of pro-fibrotic, factors, blockade of collagen synthesis, anti-inflammatory & antioxidant effects	Approved	Limited therapeutic effect, merely slowing disease progression. Severe gastrointestinal (GI) reactions, photosensitivity and rash, hepatotoxicity.	Oral	FDA/NMPA	(King et al., 2014)
Nintedanib	2014/2025	Small molecules	Nintedanib	IPF/SSc-ILD/PF-ILD	triple angiokinase inhibitor (FGFR 1–3, PDGFR α/β, VEGFR 1–3).	Approved	Low oral bioavailability, frequent GI adverse effects like diarrhea (>60% incidence), high monthly out-of-pocket expenses (∼$397.50).	Oral	FDA/NMPA	(Richeldi et al., 2014)
Nexavar®	2005/2006	Small molecules	Sorafenib	HCC/RCC/DTC	a multi-kinase inhibitor, dually blocks intracellular proliferation pathways (RAF/MEK/ERK) and cell surface angiogenic pathways (VEGFR/PDGFR).	Approved	frequently causes debilitating hand-foot skin reactions, hypertension, gastrointestinal toxicities, and bleeding risks	Oral	FDA/NMPA	(Llovet et al., 2008)
Ocaliva®	2016	Small molecules	Obeticholic acid	PBC	Farnesoid X receptor (FXR) agonist, selectively binding and activating FXRs located in hepatocytes and enterocytes.	Approved	Risk of serious and fatal liver injury, severe pruritus, lipid profile worsening.	Oral	FDA	(Tanaka et al., 2024, Wang et al., 2024)
Liraglutide	2010	Small molecules	Liraglutide	T2DM	Activates the GLP-1 receptor	Approved	The requirement for daily subcutaneous injections (which reduces patient compliance compared to weekly or oral alternatives), warning for the risk of thyroid c-cell tumors, and a potential risk of acute pancreatitis.	Subcutaneous Injection	FDA	(Armstrong et al., 2016)
Lanifibranor	N/A	Small molecule	Lanifibranor	NASH with liver fibrosis	PPARα/δ pan-agonist	Phase III (NCT04849728)	NATiV3 trial ongoing, efficacy and safety awaiting validation.	Oral	N/A	(Barb et al., 2025, Boursier et al., 2025)
Efruxifermin	N/A	Fusion protein	Efruxifermin	NASH with liver fibrosis	Fc-FGF21 fusion protein analog	Phase III	Subcutaneous injection, phase 2a showed significant liver fat reduction, long-term data needed.	Subcutaneous injection	N/A	(Kamrul-Hasan et al., 2025, Flavin, 2024)
Cilofexor	N/A	Small molecule	Cilofexor	MASH/cirrhosis	Non-steroidal FXR agonist	Phase III	Combination therapy shows high fibrosis improvement efficiency, long-term safety validation needed.	Oral	N/A	(Trauner et al., 2026, Jiang et al., 2022)
Pemvidutide	N/A	Peptide drug	Pemvidutide	MASH with F2/F3 fibrosis	GLP-1/glucagon dual agonist	Phase IIb (IMPACT)	*n* = 212, 24 weeks, 1.2 mg, 59.1% MASH resolution without fibrosis worsening, weight-loss mediated effect.	Subcutaneous injection	N/A	(Lucca Andrade et al., 2025, Noureddin et al., 2025)
Bexotegrast (PLN-74809)	N/A	Small molecule	PLN-74809	PSC with liver fibrosis	αvβ6/αvβ1 integrin dual inhibitor (TGF-β activation blockade)	Phase II complete (INTEGRIS-PSC)	*n* = 117, well tolerated, ELF score and PRO-C3 improved, rare disease (PSC) limits market size.	Oral	N/A	(Hirschfield et al., 2026)
Fazirsiran	N/A	RNAi therapeutic	siRNA targeting *Z*-AAT mRNA	AATD-associated liver fibrosis	degradation of mutant Z-AAT mRNA	Phase II (SEQUOIA)	*n* = 40, 200 mg, 94% serum Z-AAT reduction, 93% liver Z-AAT reduction, limited to AATD patients.	Subcutaneous injection	N/A	(Clark et al., 2024)
DR10624 Injection	N/A	Fusion protein/peptide	Undisclosed (GLP-1/glucagon dual agonist)	MASLD/METALD with high liver fibrosis risk	lipid-lowering + anti-inflammatory + metabolic improvement	Phase II	*n* = 96, Apr 2025–May 2026, MRI-PDFF≥10%, fibroscan 8–15 kPa, registration ongoing	Subcutaneous injection	N/A	(ianping Ianping Li et al., 2025)
BMS-986263 (ND-L02-s0201)	N/A	Lipid nanoparticle (LNP)	siRNA targeting HSP47	Moderate to extensive hepatic fibrosis (F3–F4)	Silencing of HSP47 mRNA; inhibition of collagen secretion	Phase 1b/2 (NCT02227459)	*n* = 25, safety and tolerability established, siRNA off-target effects risk, intravenous administration required	Intravenous injection	N/A	(Qosa et al., 2023, Chen et al., 2025)
Nano-curcumin	N/A	Nanoparticle (nanocrystal)	Curcumin	NAFLD-associated fibrosis (F2–F3)	Anti-inflammatory/antioxidant/anti-fibrotic	Phase II RCT complete	*n* = 55, 16 weeks, 80 mg/day, improved liver enzymes (ALT, AST, CGT, LDH) but NO significant fibrosis improvement *vs* placebo; long-term benefits unconfirmed	Oral	N/A	(Gerami et al., 2025)
Candesartan	N/A	Small molecule	Candesartan	Alcoholic liver fibrosis	Angiotensin II type 1 receptor antagonist	Phase I (NCT00990639)	Small sample size, requires Phase II validation	Oral	N/A	(Alqudah et al., 2020)
Warfarin	N/A	Small molecule	Warfarin	Liver fibrosis	Anticoagulant (vitamin K antagonist)	Phase II (NCT00180674)	Exploratory anti-fibrosis indication, bleeding risk	Oral	N/A	(Poilil Surendran et al., 2017, Dhar et al., 2010)
GR-MD-02 (Belapectin)	N/A	Monoclonal antibody	Belapectin	NASH with advanced fibrosis/cirrhosis	Galectin-3 inhibitor	Phase II (NCT02421094 / NCT02462967)	Intravenous administration inconvenient, efficacy requires Phase III validation	Intravenous injection	N/A	(Traber and Zomer, 2013)
Simtuzumab	N/A	Monoclonal antibody	Simtuzumab	NASH with advanced liver fibrosis	Anti-LOXL2 monoclonal antibody	Phase II (terminated)	*n* = 544, terminated due to lack of efficacy, Phase II trial failed	Intravenous injection	N/A	(Bell et al., 2024)
Selonsertib (GS-4997)	N/A	Small molecule	Selonsertib	NASH with fibrosis (F2–F3)	ASK1 inhibitor	Phase III (terminated)	Phase III trial terminated due to lack of efficacy, failed to meet primary endpoints	Oral	N/A	(Harrison et al., 2020)
ThermoDox	N/A	Thermosensitive liposome	Doxorubicin	Hepatocellular carcinoma	Heat-responsive release + RFA combination	Phase III (NCT02112656)	Most promising nanomedicine for commercialization, liver disease indication expanding	Intravenous injection	N/A	(Yaramiri et al., 2025, Dou et al., 2017)
Pentoxifylline	N/A	Small molecules	Pentoxifylline	Intermittent claudication caused by chronic occlusive arterial disease of the limbs	non-specifically inhibiting phosphodiesterase (PDE),potent inhibitor of TNF-α	Phase III clinical trial	Frequent dosing requirement, Gastrointestinal intolerance, mild central nervous system effects, potentially increased bleeding risk.	Oral	N/A	(Alam et al., 2017)
Elafibranor	N/A	Small molecules	Elafibranor	NASH fibrosis	PPARα/δ agonism	Phase III	Phase III RESOLVE-IT trial terminated due to failure to meet primary endpoint	Oral	N/A	(Pramanik et al., 2024, Zhang et al., 2024a)
Cenicriviroc	N/A	Small molecules	Cenicriviroc	NASH with liver fibrosis	CCR2/CCR5 antagonism	Phase III	Phase III AURORA trial terminated due to futility	Oral	N/A	(Anstee et al., 2024)
Emricasan	N/A	Small molecules	Emricasan	Liver cirrhosis / liver fibrosis	Caspase inhibition	Phase III	Clinical trial results are controversial, efficacy requires further validation.	Oral	N/A	(Frenette et al., 2021)
Irbesartan	N/A	Small molecules	AT1R blockade	Hypertension with liver fibrosis	AT1R blockade	Phase III	Phase III clinical trial in China, anti-fibrotic indication under submission.	Oral	N/A	(Schepke et al., 2008)
Ezetimibe + Simvastatin	N/A	Small molecule drug combination	Lipid-lowering + anti-inflammatory	NASH fibrosis	Lipid-lowering + anti-inflammatory	Phase II	Phase II trial showed no improvement in fibrosis	Oral	N/A	(Abel et al., 2009, Musso et al., 2011)
Obeticholic acid + atorvastatin	N/A	Combination therapy	FXR agonism + lipid-lowering	NASH with liver fibrosis	FXR agonism + lipid-lowering	Phase III	Combination therapy in phase III trial	Oral	N/A	(Pockros et al., 2019)

In 2024, Resmetirom became the first FDA-approved drug for moderate-to-advanced liver fibrosis associated with nonalcoholic steatohepatitis (NASH). However, its fibrosis reversal rate ranges merely from 25.9% to 29.9%, and its long-term safety requires further investigation (Brett, 2024; Harrison et al., 2024). In 2025, Semaglutide was approved for metabolic dysfunction-associated steatohepatitis (MASH) accompanied by stage F2 to F3 fibrosis. Nevertheless, unlike therapeutics that directly target fibrogenic pathways, its anti-fibrotic effect is primarily contingent upon weight loss (Jara et al., 2025). This indirect mechanism implies that its efficacy is highly dependent on the extent of patient weight reduction. For populations unable to achieve significant weight loss, the anti-fibrotic benefits may be quite limited. In contrast to medications still in clinical use, Ocaliva® (obeticholic acid), which was approved in 2016, was withdrawn from the market in September 2025 due to the risk of fatal liver injury (Tanaka et al., 2024; Wang et al., 2024). This event underscores that sufficient efficacy alone cannot sustain successful clinical translation; the safety of chronic administration is equally paramount.

Furthermore, the failures observed in late-stage clinical trials warrant equal attention. Selonsertib, cenicriviroc, elafibranor, and simtuzumab all advanced to phase II or phase III studies, demonstrating initial promise in preclinical and early-phase trials (Harrison et al., 2020; Anstee et al., 2024; Zhang et al., 2024a; Bell et al., 2024). However, they were ultimately terminated due to insufficient efficacy. These setbacks highlight the fundamental discrepancies between preclinical models and human patient populations. Although the standard CCl_4_-induced fibrosis model is convenient and highly reproducible, it fails to comprehensively recapitulate the metabolic, inflammatory, and genetic heterogeneity of human liver fibrosis. This limitation is particularly pronounced in MASH, where patient fibrosis develops over a decades-long chronic process against a backdrop of obesity, insulin resistance, and dyslipidemia (Vacca et al., 2024; Ning et al., 2025; Ezhilarasan et al., 2025). Additionally, the repeated failures of single-pathway inhibitors suggest robust compensatory mechanisms among fibrogenic signaling networks, making it difficult for single-target interventions to achieve optimal therapeutic outcomes. This phenomenon indicates that combination therapies or multi-target delivery systems may represent potential avenues for breaking the current translational bottleneck.

Nanomedicine offers a potential pathway for implementing more sophisticated therapeutic strategies. Nanoparticulate systems can facilitate targeted drug delivery to HSCs or other key effector cells, thereby enhancing local drug concentrations while minimizing systemic toxicity (Yang et al., 2025; Zhang et al., 2026b; Li et al., 2025). BMS-986263 is a lipid nanoparticle encapsulating HSP47 siRNA (Qosa et al., 2023). Early-phase trials have confirmed its safety and tolerability, as well as the feasibility of targeted nucleic acid delivery to the fibrotic liver. However, this agent requires intravenous administration, a route that restricts its long-term utility in managing chronic diseases. Prolonged therapy necessitates repeated infusions, resulting in poor patient compliance.

Unlike intravenously administered nucleic acid nanoparticles, oral nano-curcumin faces a different dilemma. Although nanocrystal formulations have improved its bioavailability, clinical trials have only observed improvements in enzymatic markers without achieving a significant reversal of fibrosis. This demonstrates that the optimization of delivery technologies cannot compensate for the inherent lack of efficacy of the active pharmaceutical ingredient, optimal biodistribution and intrinsic drug potency are equally critical.

Beyond small-molecule and nucleic acid drugs, certain biologics are also exploring alternative therapeutic strategies. Lanifibranor and efruxifermin, currently in phase III clinical trials, target upstream metabolic and hormonal regulators, rather than directly inhibiting collagen synthesis or HSC activation as seen with conventional anti-fibrotic agents (Barb et al., 2025; Boursier et al., 2025; Kamrul-Hasan et al., 2025; Flavin, 2024). Furthermore, bexotegrast and fazirsiran, employing precision medicine strategies, have yielded positive results in the treatment of primary sclerosing cholangitis (PSC) and alpha-1 antitrypsin deficiency (AATD), respectively (Hirschfield et al., 2026; Clark et al., 2024). The success of these narrowly targeted agents in rare diseases highlights the profound heterogeneity of liver fibrosis, underscoring that attempting to cover all patient groups with a single medication is unrealistic. Concurrently, the failure of numerous phase III trials indicates that broad NASH or MASH cohorts can easily mask efficacy signals that might only be discernible within more homogeneous subgroups (Harrison et al., 2020; Bell et al., 2024; Anstee et al., 2024; Zhang et al., 2024a; Pramanik et al., 2024).

In conclusion, the clinical translation of therapeutics for liver fibrosis continues to face severe challenges. From the limited approval of resmetirom to the termination of phase III trials for selonsertib and cenicriviroc, the field continues to navigate a path of exploratory progression. Moving forward, it is imperative to identify more precise targets, optimize combinational strategies, and establish robust patient stratification frameworks.

## 4 Challenges and outlook

### 4.1 Biological complexity and therapeutic challenges in liver fibrosis

Hitherto, considerable advancements have been made in the study of liver fibrosis pathogenesis. However, key challenges remain that need to be addressed for further exploration and understanding. Firstly, the intricacy of the pathogenic mechanisms, characterized by the interplay of HSCs activation, inflammatory mediators, immune response orchestration, and ECM remodeling, stands as a barrier to comprehensive understanding and effective therapeutic intervention (Baiocchini et al., 2016; Duarte et al., 2015; Gandhi, 2017; Hammerich and Tacke, 2023b; Roderfeld, 2018; Zhang et al., 2024c).

One of the principal challenges in this domain is the complex pathophysiological processes in the liver fibrosis. Liver fibrosis is not a consequence of a single factor but a culmination of several intricate factors such as HSCs activation, cytokine and chemokine involvement, oxidative stress, and immune responses (Gandhi, 2017; Hammerich and Tacke, 2023b; Shang et al., 2018; Tsuchida and Friedman, 2017b; Cai et al., 2021; Zhang et al., 2024c). Each of these factors presents a potential therapeutic target, but their inter-dependence makes it difficult to predict the outcomes of targeting just one pathway. Moreover, the deposition of ECM components, predominantly collagen, poses a significant impediment to drug delivery (Böttcher and Pinzani, 2017; Khalid et al., 2017). This pathological accumulation results in substantial alterations to hepatic architecture, thereby creating formidable physical barriers that disturb the effective delivery of pharmacological agents to targeted lesion areas in the liver. Overcoming this barrier is pivotal for enhancing the efficacy of existing drug delivery therapies. The reversibility of liver fibrosis, particularly delineating the threshold beyond which the condition becomes irreversible, remains an unclear aspect of the disease (Sun and Kisseleva, 2015). This ambiguity in understanding the reversible nature of early-stage fibrosis *versus* the irreversible progression in advanced stages complicates the development of targeted therapeutic strategies. Additionally, the etiological heterogeneity of liver fibrosis, attributed to diverse causes such as viral infections and chronic alcohol abuse, presents a challenge in the development of therapeutic approaches (Knolle and Thimme, 2014; Parola and Pinzani, 2019). This variability necessitates a paradigm shift toward personalized medicine, which can predicate an intricate understanding of the specific molecular and cellular mechanisms driving fibrosis in each specific case.

### 4.2 Translational barriers of nanoparticle-based antifibrotic therapies

Despite promising preclinical efficacy, the clinical translation of nanoparticle-based antifibrotic therapies remains challenging. First, large-scale manufacturing of multifunctional nanoparticles with reproducible physicochemical properties remains difficult. Parameters including particle size, zeta potential, drug loading efficiency, and surface ligand density may vary significantly during scale-up production, thereby affecting therapeutic consistency and regulatory approval (Joyce et al., 2024; Mitragotri et al., 2015).

Second, the biological distribution of nanoparticles is highly heterogeneous among patients due to variations in liver vascularization, fibrosis stage, immune status, and reticuloendothelial system activity. Excessive uptake by KCs and splenic macrophages may substantially reduce the effective accumulation of nanoparticles in aHSCs (He et al., 2024).

In addition, the immunogenicity and long-term biosafety of nanocarriers require further investigation. Repeated administration of LNPs, polymeric micelles, or extracellular vesicle-based systems may induce complement activation, accelerated blood clearance, or chronic hepatic inflammation (Lu et al., 2024; Chen et al., 2024). Furthermore, the long-term metabolism and biodegradation of inorganic nanomaterials remain insufficiently understood.

Regulatory challenges also limit clinical translation. The complexity of multifunctional nanoplatforms complicates quality control, batch reproducibility, and standardized safety evaluation (Havelikar et al., 2024). Therefore, future studies should prioritize clinically translatable nanoplatforms with simplified compositions, scalable manufacturing processes, and well-defined pharmacokinetic profiles (Tong et al., 2024).

In conclusion, future research on liver fibrosis pathogenesis should be strategically focused on understanding the complex network of pathogenic factors, designing suitable strategies to overcome ECM-induced impediments in drug delivery, clarifying reversibility of liver fibrosis, and building up novel intervention therapies based on the unique etiologies.

To achieve the above objectives, future research should be directed toward the following key areas. First, artificial intelligence-based computational design of nanocarriers should be pursued (Sahu and Satapathy, 2026). Machine learning algorithms ought to be applied to model and analyze the composition of ECM components, phenotypes of aHSCs, and intratissular drug distribution within the fibrotic microenvironment. This approach facilitates the rational design of nanoscale delivery systems capable of penetrating the ECM barrier, thereby enhancing drug delivery precision. In addition, the development of multifunctional nanoplatforms for gene-drug codelivery is warranted (Li et al., 2026; Liu et al., 2026; Zhou et al., 2022b). Nanocarriers engineered to simultaneously encapsulate anti-fibrotic small-molecule agents and gene therapeutics should be constructed, enabling multi-target synergistic strategies to counteract complex pathophysiological processes and overcome the limitations associated with single-target interventions. Furthermore, personalized anti-fibrotic nanotherapeutic strategies should be advanced (Zhang et al., 2026a; Kong et al., 2026). Based on patient etiology and fibrotic stage, a nanomedicine library matched to the specific etiology-stage profile should be established in conjunction with molecular mapping. High-throughput drug screening utilizing microfluidic chips or organ-on-a-chip models ought to be performed to tailor nanotherapies with specific targeting capabilities for individual patients, thereby effectuating a paradigm shift from generalized treatment to precision stratified therapy. Lastly, a multi-modal quantitative assessment system for determining the reversibility of liver fibrosis should be established. Data derived from elastography, ECM degradation-related molecular biomarkers, and single-cell transcriptomics should be integrated to construct a quantitative threshold model that distinguishes reversible from irreversible fibrosis (Zhang et al., 2025a; Chen et al., 2019; Sato et al., 2023). This model provides an objective basis for defining the reversible window and determining the optimal timing for therapeutic intervention.

Effectively addressing the aforementioned challenges and promoting the clinical translation of these cutting-edge technologies is of paramount importance for improving clinical outcomes in liver fibrosis.

## 5 Conclusion

Liver fibrosis, a reversible wound-healing response to chronic liver injury, poses a significant global health burden due to its progression to cirrhosis and liver failure. Current therapeutic approaches primarily target the underlying causes, such as viral hepatitis or metabolic disorders, but fail to directly reverse fibrosis or halt its progression. Advances in drug delivery systems offer promising solutions to overcome these limitations by enabling precise targeting of fibrotic tissue, enhancing drug bioavailability, and minimizing systemic side effects. Emerging technologies, including nanoparticle-based carriers, lipid-based formulations and stimuli-responsive systems, have shown potential in delivering antifibrotic agents to aHSCs and fibrotic regions with high specificity and efficiency.

Despite these advancements, several challenges remain. The dense ECM in fibrotic liver tissue presents a significant barrier to effective drug penetration. Additionally, ensuring the biocompatibility, scalability, and long-term safety of innovative drug delivery systems is critical for clinical application. Translational hurdles, such as variability in patient response and the lack of robust preclinical models that accurately mimic human liver fibrosis, also need to be addressed. Future research should focus on integrating insights from liver fibrosis pathophysiology with advanced delivery technologies to develop tailored, multifunctional therapeutic strategies. Collaboration across disciplines, coupled with innovations in precision medicine, holds the potential to significantly improve the treatment landscape for liver fibrosis, ultimately improving patient outcomes.

## Author contribution

Yibin Cai, Jiayin Li, and Shenyu Chen: Data curation and original draft preparation; Hao Li, Qin Wei, Chenshi Lin, Shuyuan Zhang and Boran Li: Writing and analysis; Hui Zhang, Jie Tan, Xiuhua You, Xuesong Zhong: Graphical Illustration of figures; Chao Qin, Chao Teng and Jie Liu: Conceptualization, Supervision, Manuscript Editing and Review.

## CRediT authorship contribution statement

**Yibin Cai:** Writing – review & editing, Writing – original draft, Supervision, Funding acquisition, Conceptualization. **Jiayin Li:** Writing – original draft, Formal analysis, Conceptualization. **Shenyu Chen:** Writing – original draft, Conceptualization. **Hui Zhang:** Writing – original draft, Visualization, Formal analysis, Conceptualization. **Jie Tan:** Writing – original draft, Formal analysis, Conceptualization. **Hao Li:** Writing – original draft, Formal analysis, Conceptualization. **Shuyuan Zhang:** Writing – original draft, Visualization, Formal analysis. **Boran Li:** Writing – original draft, Visualization, Formal analysis. **Qin Wei:** Writing – original draft, Visualization, Formal analysis. **Chenshi Lin:** Writing – original draft, Visualization, Formal analysis. **Xuesong Zhong:** Writing – original draft, Visualization, Formal analysis. **Xiuhua You:** Writing – original draft, Formal analysis. **Chao Qin:** Writing – review & editing, Writing – original draft, Software, Conceptualization. **Chao Teng:** Writing – review & editing, Writing – original draft, Supervision, Funding acquisition. **Jie Liu:** Writing – review & editing, Writing – original draft, Supervision, Conceptualization.

## Consent for publication

N/A.

## Ethics approval and consent to participate

N/A.

## Funding

This study was supported by the NSFC82400095, 10.13039/100003536Youth Foundation of Jiangsu Province (BK20231009), Jiangsu Funding Program for Excellent Postdoctoral Talent (2022ZB313), 10.13039/501100003392Natural Science Foundation of Fujian Province of China (2025J01706, 2025J011164), 10.13039/501100013795Research Foundation for Advanced Talents of Fujian Medical University (XN240017), Fujian Provincial Health Technology Project (2025QNA052), 10.13039/501100013795Startup Fund for Scientific Research of Fujian Medical University (2018QH1013).

## Declaration of competing interest

The authors declare that they have no known competing financial interests or personal relationships that could have appeared to influence the work reported in this paper.

## Data Availability

The datasets analyzed during the current study are available from the corresponding author on reasonable request.

## References

[bb0005] Aashaq S., Batool A., Mir S.A. (2022). TGF-β signaling: a recap of SMAD-independent and SMAD-dependent pathways. J. Cell. Physiol..

[bb0010] Abel T., Fehér J., Dinya E. (2009). Safety and efficacy of combined ezetimibe/simvastatin treatment and simvastatin monotherapy in patients with non-alcoholic fatty liver disease. Med. Sci. Monit..

[bb0015] Acharya P., Chouhan K., Weiskirchen S. (2021).

[bb0020] Alam S., Nazmul Hasan S., Mustafa G. (2017). Effect of pentoxifylline on histological activity and fibrosis of nonalcoholic steatohepatitis patients: a one year randomized control trial. J. Transl. Intern. Med..

[bb0025] Alqudah M., Hale T.M., Czubryt M.P. (2020). Targeting the renin-angiotensin-aldosterone system in fibrosis. Matrix Biol..

[bb0030] Andrade R.J., Chalasani N., Björnsson E.S. (2019). Drug-induced liver injury. Nat. Rev. Dis. Primers.

[bb0035] Anstee Q.M., Neuschwander-Tetri B.A., Wai-Sun Wong V. (2024). Cenicriviroc lacked efficacy to treat liver fibrosis in nonalcoholic steatohepatitis: AURORA phase III randomized study. Clin. Gastroenterol. Hepatol..

[bb0040] Araujo David B., Andreata F., Blériot C. (2026). Kupffer cells in liver homeostasis and disease: from immune sentinels to metabolic gatekeepers. Nat. Rev. Immunol..

[bb0045] Armillotta Maria Giovanna, Nbsp Lara Lizzi (2025). Nanoparticle-based systems for liver therapy: Overcoming fibrosis and enhancing drug efficacy.

[bb0050] Armstrong M.J., Gaunt P., Aithal G.P. (2016). Liraglutide safety and efficacy in patients with non-alcoholic steatohepatitis (LEAN): a multicentre, double-blind, randomised, placebo-controlled phase 2 study. Lancet.

[bb0055] Arpino V., Brock M., Gill S.E. (2015). The role of TIMPs in regulation of extracellular matrix proteolysis. Matrix Biol..

[bb0060] Arseni L., Lombardi A., Orioli D. (2018). From structure to phenotype: impact of collagen alterations on human health. Int. J. Mol. Sci..

[bb0065] Baarsma H.A., Menzen M.H., Halayko A.J. (2011). β-Catenin signaling is required for TGF-β1-induced extracellular matrix production by airway smooth muscle cells. Am. J. Phys. Lung Cell. Mol. Phys..

[bb0070] Baghaei K., Mazhari S., Tokhanbigli S. (2022). Therapeutic potential of targeting regulatory mechanisms of hepatic stellate cell activation in liver fibrosis. Drug Discov. Today.

[bb0075] Baiocchini A., Montaldo C., Conigliaro A. (2016). Extracellular matrix molecular remodeling in human liver fibrosis evolution. PLoS One.

[bb0080] Barb D., Kalavalapalli S., Godinez Leiva E. (2025). Pan-PPAR agonist lanifibranor improves insulin resistance and hepatic steatosis in patients with T2D and MASLD. J. Hepatol..

[bb0085] Bataller R., Brenner D.A. (2005). Liver fibrosis. J. Clin. Invest..

[bb0090] Baumann B., Hayashida T., Liang X. (2016). Hypoxia-inducible factor-1alpha promotes glomerulosclerosis and regulates COL1A2 expression through interactions with Smad3. Kidney Int..

[bb0095] Beljaars L., Olinga P., Molema G. (2001). Characteristics of the hepatic stellate cell-selective carrier mannose 6-phosphate modified albumin (M6P(28)-HSA). Liver.

[bb0100] Bell J.A., Davies E.R., Brereton C.J. (2024). Spatial transcriptomic validation of a biomimetic model of fibrosis enables re-evaluation of a therapeutic antibody targeting LOXL2. Cell Rep. Med..

[bb0105] Bobowski-Gerard M., Zummo F.P., Staels B. (2018). Retinoids issued from hepatic stellate cell lipid droplet loss as potential signaling molecules orchestrating a multicellular liver injury response. Cells.

[bb0110] Böttcher K., Pinzani M. (2017). Pathophysiology of liver fibrosis and the methodological barriers to the development of anti-fibrogenic agents. Adv. Drug Deliv. Rev..

[bb0115] Boursier J., Hervé H., Roux M. (2025). Biomarkers of histological response in Lanifibranor-treated patients with metabolic dysfunction-associated steatohepatitis. Clin. Gastroenterol. Hepatol..

[bb0120] Brett A. (2024). Resmetirom, the first drug approved by the U.S. FDA for treating patients with nonalcoholic steatohepatitis. NEJM J. Watch.

[bb0125] Cai J., Hu M., Chen Z. (2021). The roles and mechanisms of hypoxia in liver fibrosis. J. Transl. Med..

[bb0130] Canestrale A.R., Kholia S., Dimuccio V. (2025). Targeted hepatic delivery of bioactive molecules via nanovesicles: recent developments and emerging directions. J. Pers. Med..

[bb0135] Canestrale A.R., Kholia S., Dimuccio V. (2025).

[bb0140] Chai N.-L., Fu Q., Shi H. (2012). Oxymatrine liposome attenuates hepatic fibrosis via targeting hepatic stellate cells. World J. Gastroenterol..

[bb0145] Chen Y., Huang Y., Reiberger T. (2014). Differential effects of sorafenib on liver versus tumor fibrosis mediated by stromal-derived factor 1 alpha/C-X-C receptor type 4 axis and myeloid differentiation antigen–positive myeloid cell infiltration in mice. Hepatology.

[bb0150] Chen W., Yan X., Xu A. (2019). Dynamics of elastin in liver fibrosis: accumulates late during progression and degrades slowly in regression. J. Cell. Physiol..

[bb0155] Chen Q., Huang J., Ye Y. (2023). Delivery of hydroxycamptothecin via sonoporation: an effective therapy for liver fibrosis. J. Control. Release.

[bb0160] Chen L., Guo W., Mao C. (2024). Liver fibrosis: pathological features, clinical treatment and application of therapeutic nanoagents. J. Mater. Chem. B.

[bb0165] Chen Y.N., Li M.Q., Zhang H.J. (2025). Nanoparticle-based drug delivery systems: a promising approach for the treatment of liver fibrosis. Int. J. Pharm. X.

[bb0170] Chen X., Zhang J., Guo L. (2026). Decoding organ fibrosis: mechanistic insights and emerging therapeutic strategies. Signal Transduct. Target. Ther..

[bb0175] Cheng W.Y., Zeng X.X., Cheng P. (2024). Loureirin B ameliorates cholestatic liver fibrosis via AKT/mTOR/ATG7-mediated autophagy of hepatic stellate cells. Eur. J. Pharmacol..

[bb0180] Cho Y., Szabo G. (2021). Two faces of neutrophils in liver disease development and progression. Hepatology.

[bb0185] Chung S., Moon H., Ju H.-L. (2016). Hepatic expression of Sonic Hedgehog induces liver fibrosis and promotes hepatocarcinogenesis in a transgenic mouse model. J. Hepatol..

[bb0190] Chung S., Moon H., Ju H.L. (2016). Hepatic expression of Sonic Hedgehog induces liver fibrosis and promotes hepatocarcinogenesis in a transgenic mouse model. J. Hepatol..

[bb0195] Clark V.C., Strange C., Strnad P. (2024). Fazirsiran for adults with Alpha-1 Antitrypsin Deficiency Liver Disease: a phase 2 placebo controlled trial (SEQUOIA). Gastroenterology.

[bb0200] Colino C., Lanao J.M., Gutierrez-Millan C. (2020). Targeting of hepatic macrophages by therapeutic nanoparticles. Front. Immunol..

[bb0205] Deng X., Li Z., Xu Z. (2026). CAT1 modulates hepatic fibrosis via IL-6-mediated inflammatory and fibrogenic pathways in hepatic stellate cells. Biochim. Biophys. Acta (BBA) - Mol. Cell Res..

[bb0210] Devarbhavi H., Asrani S.K., Arab J.P. (2023). Global burden of liver disease: 2023 update. J. Hepatol..

[bb0215] Dewidar B., Meyer C., Dooley S. (2019). TGF-β in hepatic stellate cell activation and liver fibrogenesis—updated 2019. Cells.

[bb0220] Dhar A., Anstee Q., Cobbold J. (2010). P17 Anticoagulation for liver fibrosis: a pilot study in hepatitis C infected patients.

[bb0225] Dixon L.J., Barnes M., Tang H. (2013). Kupffer cells in the liver. Compr. Physiol..

[bb0230] Dong H., Guo W., Zhou Z. (2026).

[bb0235] Dou Y., Hynynen K., Allen C. (2017). To heat or not to heat: challenges with clinical translation of thermosensitive liposomes. J. Control. Release.

[bb0240] Doumpas N., Lampart F., Robinson M.D. (2019). TCF/LEF dependent and independent transcriptional regulation of Wnt/β-catenin target genes. EMBO J..

[bb0245] Duan B.W., Liu Y.J., Li X.N. (2023). An autologous macrophage-based phenotypic transformation-collagen degradation system treating advanced liver fibrosis. Adv. Sci. (Weinh).

[bb0250] Duarte S., Baber J., Fujii T. (2015). Matrix metalloproteinases in liver injury, repair and fibrosis. Matrix Biol..

[bb0255] Ebrahimi H., Naderian M., Sohrabpour A.A. (2018). New concepts on reversibility and targeting of liver fibrosis; a review article. Middle East J. Dig. Dis..

[bb0260] Eftekhari A., Arjmand A., Asheghvatan A. (2021). The potential application of magnetic nanoparticles for liver fibrosis theranostics. Front. Chem..

[bb0265] El Andaloussi S., Mäger I., Breakefield X.O. (2013). Extracellular vesicles: biology and emerging therapeutic opportunities. Nat. Rev. Drug Discov..

[bb0270] Ezhilarasan D., Karthikeyan S., Najimi M. (2025). Preclinical liver toxicity models: advantages, limitations and recommendations. Toxicology.

[bb0275] Fabregat I., Moreno-Càceres J., Sánchez A. (2016). TGF-β signalling and liver disease. FEBS J..

[bb0280] Fan Q.-Q., Zhang C.-L., Qiao J.-B. (2020). Extracellular matrix-penetrating nanodrill micelles for liver fibrosis therapy. Biom. J..

[bb0285] Finnson K.W., Almadani Y., Philip A. (2020). Seminars in Cell & Developmental Biology.

[bb0290] Flavin B. (2024). Nonalcoholic steatohepatitis/metabolic dysfunction-associated steatohepatitis emerging market: preparing managed care for early intervention, equitable access, and integrating the patient perspective. J. Manag. Care Spec. Pharm..

[bb0295] Foglia B., Cannito S., Bocca C. (2019). ERK pathway in activated, myofibroblast-like, hepatic stellate cells: a critical signaling crossroad sustaining liver fibrosis. Int. J. Mol. Sci..

[bb0300] Frenette C., Kayali Z., Mena E. (2021). Emricasan to prevent new decompensation in patients with NASH-related decompensated cirrhosis. J. Hepatol..

[bb0305] Fruman D.A., Chiu H., Hopkins B.D. (2017). The PI3K pathway in human disease. Cell.

[bb0310] Gan C., Yuan Y., Shen H. (2025). Liver diseases: epidemiology, causes, trends and predictions. Signal Transduct. Target. Ther..

[bb0315] Gandhi C.R. (2017). Hepatic stellate cell activation and pro-fibrogenic signals. J. Hepatol..

[bb0320] Gao L., Zhang Z., Zhang P. (2018). Role of canonical Hedgehog signaling pathway in liver. Int. J. Biol. Sci..

[bb0325] Gao Y., Fan S., Zhao P. (2023). Β-Catenin/TCF4 inhibitors ICG-001 and LF3 alleviate BDL-induced liver fibrosis by suppressing LECT2 signaling. Chem. Biol. Interact..

[bb0330] Geervliet E., Moreno S., Baiamonte L. (2021). Matrix metalloproteinase-1 decorated polymersomes, a surface-active extracellular matrix therapeutic, potentiates collagen degradation and attenuates early liver fibrosis. J. Control. Release.

[bb0335] Gerami H., Mozaffari-Khosravi H., Mansour A. (2025). Effect of nano-curcumin supplementation on liver fibrosis in patients with NAFLD-associated fibrosis: a double-blind randomized controlled trial. Sci. Rep..

[bb0340] Gu J., Sun J., Tian K. (2023). Reversal of hepatic fibrosis by the co-delivery of drug and ribonucleoprotein-based genome editor. Biom. J..

[bb0345] Gupta M.K., Vadde R. (2025). TLR-based therapeutic strategies for hepatocellular carcinoma. Cytokine Growth Factor Rev..

[bb0350] Gupta G., Khadem F., Uzonna J.E. (2019). Role of hepatic stellate cell (HSC)-derived cytokines in hepatic inflammation and immunity. Cytokine.

[bb0355] Györfi A.H., Matei A.-E., Distler J.H. (2018). Targeting TGF-β signaling for the treatment of fibrosis. Matrix Biol..

[bb0360] Hammerich L., Tacke F. (2023). Hepatic inflammatory responses in liver fibrosis. Nat. Rev. Gastroenterol. Hepatol..

[bb0365] Hammerich L., Tacke F. (2023). Hepatic inflammatory responses in liver fibrosis. Nat. Rev. Gastroenterol. Hepatol..

[bb0370] Han X., Gong N., Xue L. (2023). Ligand-tethered lipid nanoparticles for targeted RNA delivery to treat liver fibrosis. Nat. Commun..

[bb0375] Han-Jing Zhangdi S.-B.S., Wang Fei, Liang Zi-Yu, Yan Yu-Dong, Qin Shan-Yu, Jiang Hai-Xing (2019). Crosstalk network among multiple inflammatory mediators in liver fibrosis. World J. Gastroenterol..

[bb0380] Hao Y., Song K., Tan X. (2022). Reactive oxygen species-responsive polypeptide drug delivery system targeted activated hepatic stellate cells to ameliorate liver fibrosis. ACS Nano.

[bb0385] Harrison S.A., Wong V.W., Okanoue T. (2020). Selonsertib for patients with bridging fibrosis or compensated cirrhosis due to NASH: results from randomized phase III STELLAR trials. J. Hepatol..

[bb0390] Harrison S.A., Bedossa P., Guy C.D. (2024). A phase 3, randomized, controlled trial of resmetirom in NASH with liver fibrosis. N. Engl. J. Med..

[bb0395] Hata A., Chen Y.-G. (2016). TGF-β signaling from receptors to Smads. Cold Spring Harb. Perspect. Biol..

[bb0400] Havelikar U., Ghorpade K.B., Kumar A. (2024). Comprehensive insights into mechanism of nanotoxicity, assessment methods and regulatory challenges of nanomedicines. Discover Nano.

[bb0405] He Y., Wang Y., Wang L. (2024). Understanding nanoparticle-liver interactions in nanomedicine. Expert Opin. Drug Deliv..

[bb0410] Hegazy Rabab A.-M. (2025). Unraveling liver cirrhosis: bridging pathophysiology to innovative therapeutics. J. Gastroenterol. Hepatol..

[bb0415] Hirschfield G.M., Kowdley K.V., Trivedi P.J. (2026). Phase II INTEGRIS-PSC trial of bexotegrast, an α(v)β(6)/α(v)β(1) integrin inhibitor, in primary sclerosing cholangitis. J. Hepatol..

[bb0420] Hou L.S., Zhai X.P., Zhang Y.W. (2024). Targeted inhibition of autophagy in hepatic stellate cells by hydroxychloroquine: an effective therapeutic approach for the treatment of liver fibrosis. Liver Int..

[bb0425] Hove M.T., Smyris A., Booijink R. (2024). Engineered SPIONs functionalized with endothelin a receptor antagonist ameliorate liver fibrosis by inhibiting hepatic stellate cell activation. Bioact. Mater..

[bb0430] Hu H.-H., Chen D.-Q., Wang Y.-N. (2018). New insights into TGF-β/Smad signaling in tissue fibrosis. Chem. Biol. Interact..

[bb0435] Hu X., Ge Q., Zhang Y. (2023). A review of the effect of exosomes from different cells on liver fibrosis. Biomed. Pharmacother..

[bb0440] Hua Y., Yang Y., Li Q. (2018). Oligomerization of Frizzled and LRP5/6 protein initiates intracellular signaling for the canonical WNT/β-catenin pathway. J. Biol. Chem..

[bb0445] Huang P., Yan R., Zhang X. (2019). Activating Wnt/β-catenin signaling pathway for disease therapy: challenges and opportunities. Pharmacol. Therapeut..

[bb0450] Huang E., Peng N., Xiao F. (2020). The roles of immune cells in the pathogenesis of fibrosis. Int. J. Mol. Sci..

[bb0455] Huang J., Huang H., Wang Y. (2023). Retinol-binding protein-hijacking nanopolyplex delivering siRNA to cytoplasm of hepatic stellate cell for liver fibrosis alleviation. Biom. J..

[bb0460] Huang T., Shang Z., Nie L. (2025). m6A modified ATG9A is required in regulating autophagy to promote HSCs activation and liver fibrosis. Cell. Signal..

[bb0465] Ianping Li M.D., Fang Yongliang (2025). Late-breaking science abstracts and featured science abstracts from the American Heart Association’s Scientific Sessions 2025 and late-breaking abstracts in resuscitation science from the Resuscitation Science Symposium 2025. Circulation.

[bb0470] Ibrahim M.A., Othman R., Chee C.F. (2023).

[bb0475] Jara M., Norlin J., Kjær M.S. (2025). Modulation of metabolic, inflammatory and fibrotic pathways by semaglutide in metabolic dysfunction-associated steatohepatitis. Nat. Med..

[bb0480] Jiang L., Liu X., Wei H. (2022). Structural insight into the molecular mechanism of cilofexor binding to the farnesoid X receptor. Biochem. Biophys. Res. Commun..

[bb0485] Jin Y., Wang H., Yi K. (2020). Applications of nanobiomaterials in the therapy and imaging of acute liver failure. Nano Micro Lett..

[bb0490] Joyce P., Allen C.J., Alonso M.J. (2024). A translational framework to DELIVER nanomedicines to the clinic. Nat. Nanotechnol..

[bb0495] Kamm D.R., Mccommis K.S. (2022). Hepatic stellate cells in physiology and pathology. J. Physiol..

[bb0500] Kamrul-Hasan A.B.M., Borozan S., Jena S. (2025). Safety and efficacy of efruxifermin in metabolic dysfunction-associated steatohepatitis: a systematic review. World J. Gastrointest. Pharmacol. Ther..

[bb0505] Kaps L., Leber N., Klefenz A. (2020). In vivo siRNA delivery to immunosuppressive liver macrophages by α-mannosyl-functionalized cationic nanohydrogel particles.

[bb0510] Kaps L., Huppertsberg A., Choteschovsky N. (2022). pH-degradable, bisphosphonate-loaded nanogels attenuate liver fibrosis by repolarization of M2-type macrophages. Proc. Natl. Acad. Sci. USA.

[bb0515] Kaps L., Limeres M.J., Schneider P. (2023). Liver cell type-specific targeting by nanoformulations for therapeutic applications. Int. J. Mol. Sci..

[bb0520] Kaushal S., Annamali M., Blomenkamp K. (2010). Rapamycin reduces intrahepatic alpha-1-antitrypsin mutant Z protein polymers and liver injury in a mouse model. Exp. Biol. Med. (Maywood).

[bb0525] Khalid A., Persano S., Shen H. (2017). Strategies for improving drug delivery: nanocarriers and microenvironmental priming. Expert Opin. Drug Deliv..

[bb0530] Khatun M., Ray R.B. (2019). Mechanisms underlying hepatitis C virus-associated hepatic fibrosis. Cells.

[bb0535] Khomich O., Ivanov A.V., Bartosch B. (2019). Metabolic hallmarks of hepatic stellate cells in liver fibrosis. Cells.

[bb0540] Kim S.Y., Seki E. (2020). The Liver: Biology and Pathobiology.

[bb0545] Kim J.G., Kim M.J., Choi W.J. (2017). Wnt3A induces GSK-3β phosphorylation and β-catenin accumulation through RhoA/ROCK. J. Cell. Physiol..

[bb0550] Kim J.K., Han N.R., Park S.M. (2020). Hemistepsin a alleviates liver fibrosis by inducing apoptosis of activated hepatic stellate cells via inhibition of nuclear factor-κB and Akt. Food Chem. Toxicol..

[bb0555] Kim J., Lee C., Shin Y. (2021). sEVs from tonsil-derived mesenchymal stromal cells alleviate activation of hepatic stellate cells and liver fibrosis through miR-486-5p. Mol. Ther..

[bb0560] Kim K.R., Kim J., Back J.H. (2022). Cholesterol-mediated seeding of protein corona on DNA nanostructures for targeted delivery of oligonucleotide therapeutics to treat liver fibrosis. ACS Nano.

[bb0565] King T.E., Bradford W.Z., Castro-Bernardini S. (2014). A phase 3 trial of pirfenidone in patients with idiopathic pulmonary fibrosis.

[bb0570] Kisseleva T., Brenner D. (2021). Molecular and cellular mechanisms of liver fibrosis and its regression. Nat. Rev. Gastroenterol. Hepatol..

[bb0575] Kisseleva T., Brenner D. (2021). Molecular and cellular mechanisms of liver fibrosis and its regression. Nat. Rev. Gastroenterol. Hepatol..

[bb0580] Klepfish M., Gross T., Vugman M. (2020). LOXL2 inhibition paves the way for macrophage-mediated collagen degradation in liver fibrosis. Front. Immunol..

[bb0585] Knolle P.A., Thimme R. (2014). Hepatic immune regulation and its involvement in viral hepatitis infection. Gastroenterology.

[bb0590] Kong D., Yu S., Heegsma J. (2026). High-throughput human microfluidic organoid-on-a-chip platform for modeling liver diseases and screening nanotherapeutics. J.

[bb0595] Kostallari E., Wei B., Sicard D. (2022). Stiffness is associated with hepatic stellate cell heterogeneity during liver fibrosis. Am. J. Physiol. Gastrointest. Liver Physiol..

[bb0600] Krenkel O., Puengel T., Govaere O. (2018). Therapeutic inhibition of inflammatory monocyte recruitment reduces steatohepatitis and liver fibrosis. Hepatology.

[bb0605] Kumar V., Singh A., Gautam A.K. (2026). Dual ligand grafted liposomes for CD44 and CD206 targeted delivery of niclosamide against bisphenol A-induced hepatic fibrosis in albino Wistar rats: in vivo therapeutic efficacy and molecular pathway modulation. Naunyn Schmiedeberg’s Arch.

[bb0610] Kundu S., Chauhan S., Dhar K. (2025). Chats and spats of autophagy and innate immune systems. J. Mol. Biol..

[bb0615] Lachowski D., Cortes E., Rice A. (2019). Matrix stiffness modulates the activity of MMP-9 and TIMP-1 in hepatic stellate cells to perpetuate fibrosis. Sci. Rep..

[bb0620] Lai Q., Li W., Hu D. (2024). Hepatic stellate cell-targeted chemo-gene therapy for liver fibrosis using fluorinated peptide-lipid hybrid nanoparticles. J. Control. Release.

[bb0625] Lau H., Hu K.-Q. (2024). S1764 Risk factors, clinical presentation and non-invasive liver fibrosis tests for advanced liver fibrosis in biopsy diagnosed metabolic dysfunction-associated steatotic liver disease or steatohepatitis. Am. J. Gastroenterol..

[bb0630] Le T.V., Dinh N.B.T., Dang M.T. (2022). Effects of autophagy inhibition by chloroquine on hepatic stellate cell activation in CCl4-induced acute liver injury mouse model. J. Gastroenterol. Hepatol..

[bb0635] Le M.H., Le D.M., Baez T.C. (2024). Global incidence of adverse clinical events in non-alcoholic fatty liver disease: a systematic review and meta-analysis. Clin. Mol. Hepatol..

[bb0640] Lee J., Byun J., Shim G. (2022). Fibroblast activation protein activated antifibrotic peptide delivery attenuates fibrosis in mouse models of liver fibrosis. Nat. Commun..

[bb0645] Levada K., Omelyanchik A., Rodionova V. (2019). Magnetic-assisted treatment of liver fibrosis. Cells.

[bb0650] Li J., Chen C., Xia T. (2022). Understanding nanomaterial-liver interactions to facilitate the development of safer nanoapplications. Adv. Mater..

[bb0655] Li Y., Fan W., Link F. (2022). Transforming growth factor β latency: a mechanism of cytokine storage and signalling regulation in liver homeostasis and disease. JHEP Rep. Innov. Hepatol..

[bb0660] Li B., Huang Y., Bao J. (2023). Supramolecular nanoarchitectonics based on antagonist peptide self-assembly for treatment of liver fibrosis. Small.

[bb0665] Li F., Cheng Z., Sun J. (2023). The combination of sinusoidal perfusion enhancement and apoptosis inhibition by Riociguat plus a galactose-PEGylated bilirubin multiplexing nanomedicine ameliorates liver fibrosis progression. Nano Lett..

[bb0670] Li H., Liu T., Yang Y. (2023). Interplays of liver fibrosis-associated microRNAs: molecular mechanisms and implications in diagnosis and therapy. Genes Dis..

[bb0675] Li R., Zhang J., Liu Q. (2023). CREKA-modified liposomes target activated hepatic stellate cells to alleviate liver fibrosis by inhibiting collagen synthesis and angiogenesis. Acta Biomater..

[bb0680] Li R., Tai Y., Zhang X. (2025). Tissue-microenvironment-responsive self-assembling peptide nanoshells boost pirfenidone efficacy in the treatment of liver fibrosis. Adv. Healthc. Mater..

[bb0685] Li M., Kong X., Bai W. (2026). Bioactive lipid nanoparticles as active rehabilitative regulators for fibrotic microenvironment remodeling. Mater. Today.

[bb0690] Liang Y., Wang J., Xu C. (2023). Remodeling collagen microenvironment in liver using a biomimetic nano-regulator for reversal of liver fibrosis. Adv. Sci. (Weinh).

[bb0695] Lin C.-W., Nocka L.M., Stinger B.L. (2022). A two-component protein condensate of the EGFR cytoplasmic tail and Grb2 regulates Ras activation by SOS at the membrane. Proc. Natl. Acad. Sci. USA.

[bb0700] Lin Y., Dong M.Q., Liu Z.M. (2022). A strategy of vascular-targeted therapy for liver fibrosis. Hepatology.

[bb0705] Lin Z., Yang M., Yu X. (2024). Ponatinib alleviates non-alcoholic steatohepatitis through TFEB-mediated autophagy. Front. Pharmacol..

[bb0710] Liu P., De La Vega M.R., Dodson M. (2019). Spermidine confers liver protection by enhancing NRF2 signaling through a MAP1S-mediated noncanonical mechanism. Hepatology.

[bb0715] Liu K., Wang F.-S., Xu R. (2021). Neutrophils in liver diseases: pathogenesis and therapeutic targets. Cell. Mol. Immunol..

[bb0720] Liu M.X., Xu L., Cai Y.T. (2023). Carbon nitride-based siRNA vectors with self-produced O2 effects for targeting combination therapy of liver fibrosis via HIF-1α-mediated TGF-β1/Smad pathway. Adv. Healthc. Mater..

[bb0725] Liu Y., Zheng Y., Yang Y. (2023). Exosomes in liver fibrosis: the role of modulating hepatic stellate cells and immune cells, and prospects for clinical applications. Front. Immunol..

[bb0730] Liu Y., Zheng Y., Yang Y. (2023).

[bb0735] Liu J., Liu J., Mu W. (2024). Delivery strategy to enhance the therapeutic efficacy of liver fibrosis via nanoparticle drug delivery systems. ACS Nano.

[bb0740] Liu C., Yang H., Liu Y. (2025). Cell-specific roles of autophagy in liver fibrosis: implications for targeted pharmacotherapy. Ann. Med..

[bb0745] Liu X.Y., Mao H.Y., Hu J.S. (2025). Reactive oxygen species-responsive micelles targeting activated hepatic stellate cells for treating liver fibrosis. J. Control. Release.

[bb0750] Liu Z., Yao Z., Yang H. (2025). Leveraging the dual role of ROS in liver diseases with nanomaterials: clearing and amplifying for therapy. Nanoscale.

[bb0755] Liu M.-X., Zhu Y.-Q., Yang Y. (2026). Macrophage membrane-coated NIR light-photodegradable carbon nitride-based gene vectors for gas-gene therapy of liver fibrosis. Biom. J..

[bb0760] Llovet J.M., Ricci S., Mazzaferro V. (2008). Sorafenib in advanced hepatocellular carcinoma.

[bb0765] Lu X., Fan S., Cao M. (2024). Extracellular vesicles as drug delivery systems in therapeutics: current strategies and future challenges. J. Pharm. Investig..

[bb0770] Lua I., Li Y., Zagory J.A. (2016). Characterization of hepatic stellate cells, portal fibroblasts, and mesothelial cells in normal and fibrotic livers. J. Hepatol..

[bb0775] Luangmonkong T., Parichatikanond W., Olinga P. (2023). Targeting collagen homeostasis for the treatment of liver fibrosis: opportunities and challenges. Biochem. Pharmacol..

[bb0780] Lucca Andrade M., Suschak J.J., Georges B. (2025). 778-P: Pemvidutide, a balanced GLP-1/glucagon dual receptor agonist, enhances reverse cholesterol transport in a Golden Syrian hamster model. Diabetes.

[bb0785] Luo K. (2017). Signaling cross talk between TGF-β/Smad and other signaling pathways. Cold Spring Harb. Perspect. Biol..

[bb0790] Luo S., Yang Y., Zhao T. (2023). Albumin-based silibinin nanocrystals targeting activated hepatic stellate cells for liver fibrosis therapy. ACS Appl. Mater. Interfaces.

[bb0795] Lybrand D.B., Naiman M., Laumann J.M. (2019). Destruction complex dynamics: Wnt/β-catenin signaling alters Axin-GSK3β interactions in vivo. Development.

[bb0800] Macdonald B.T., He X. (2012). Frizzled and LRP5/6 receptors for Wnt/β-catenin signaling. Cold Spring Harb. Perspect. Biol..

[bb0805] Machado M.V., Diehl A.M. (2018). Hedgehog signalling in liver pathophysiology. J. Hepatol..

[bb0810] Manou D., Caon I., Bouris P. (2019). The Extracellular Matrix: Methods and Protocols.

[bb0815] Mejias M., Gallego J., Naranjo-Suarez S. (2020). CPEB4 increases expression of PFKFB3 to induce glycolysis and activate mouse and human hepatic stellate cells, promoting liver fibrosis. Gastroenterology.

[bb0820] Michalopoulos G.K. (2017). Hepatostat: liver regeneration and normal liver tissue maintenance. Hepatology.

[bb0825] Mitragotri S., Anderson D.G., Chen X. (2015). Accelerating the translation of nanomaterials in biomedicine. ACS Nano.

[bb0830] Mooli R.G.R., Mukhi D., Ramakrishnan S.K. (2022). Oxidative stress and redox signaling in the pathophysiology of liver diseases. Compr. Physiol..

[bb0835] Munsterman D., Kendall T.J., Khelil N. (2018). Extracellular matrix components indicate remodelling activity in different fibrosis stages of human non-alcoholic fatty liver disease. Histopathology.

[bb0840] Musso G., Cassader M., Gambino R. (2011). Cholesterol-lowering therapy for the treatment of nonalcoholic fatty liver disease: an update. Curr. Opin. Lipidol..

[bb0845] Niederreiter L., Tilg H. (2018). Cytokines and fatty liver diseases. Liver Res..

[bb0850] Nimni M.E., Harkness R.D. (2018). Collagen.

[bb0855] Ning M., Lu D., Teng B. (2025). Comprehensive study of the murine MASH models’ applicability by comparing human liver transcriptomes. Life Sci..

[bb0860] Noureddin M., Harrison S.A., Loomba R. (2025). Safety and efficacy of weekly pemvidutide versus placebo for metabolic dysfunction-associated steatohepatitis (IMPACT): 24-week results from a multicentre, randomised, double-blind, phase 2b study. Lancet (London, England).

[bb0865] Nyström H. (2021). Seminars in Cancer Biology.

[bb0870] Ortiz C., Schierwagen R., Schaefer L. (2021). Extracellular matrix remodeling in chronic liver disease. Curr. Tissue Microenviron. Rep..

[bb0875] Ostrem J.M., Peters U., Sos M.L. (2013). K-Ras(G12C) inhibitors allosterically control GTP affinity and effector interactions. Nature.

[bb0880] Pan R.L., Xiang L.X., Wang P. (2015). Low-molecular-weight fibroblast growth factor 2 attenuates hepatic fibrosis by epigenetic down-regulation of Delta-like1. Hepatology.

[bb0885] Parola M., Pinzani M. (2019). Liver fibrosis: pathophysiology, pathogenetic targets and clinical issues. Mol. Asp. Med..

[bb0890] Peeters G., Debbaut C., Friebel A. (2018). Quantitative analysis of hepatic macro- and microvascular alterations during cirrhogenesis in the rat. J. Anat..

[bb0895] Peng D., Fu M., Wang M. (2022). Targeting TGF-β signal transduction for fibrosis and cancer therapy. Mol. Cancer.

[bb0900] Perugorria M.J., Olaizola P., Labiano I. (2019). Wnt–β-catenin signalling in liver development, health and disease. Nat. Rev. Gastroenterol. Hepatol..

[bb0905] Pockros P.J., Fuchs M., Freilich B. (2019). CONTROL: a randomized phase 2 study of obeticholic acid and atorvastatin on lipoproteins in nonalcoholic steatohepatitis patients. Liver Int..

[bb0910] Poilil Surendran S., George Thomas R., Moon M.J. (2017). Nanoparticles for the treatment of liver fibrosis. Int. J. Nanomedicine.

[bb0915] Pramanik S., Pal P., Ray S. (2024). Non-alcoholic fatty liver disease in type 2 diabetes: emerging evidence of benefit of peroxisome proliferator-activated receptors agonists and incretin-based therapies. World J. Methodol..

[bb0920] Qiao J.B., Fan Q.Q., Zhang C.L. (2020). Hyperbranched lipoid-based lipid nanoparticles for bidirectional regulation of collagen accumulation in liver fibrosis. J. Control. Release.

[bb0925] Qiu Z., Milichko V.A., Zhou Y. (2023). Kupffer cell teleportation” strategy for liver fibrosis alleviation based on hybrid polymer- bimetallic sequential delivery system. Adv. Funct. Mater..

[bb0930] Qosa H., De Oliveira C., Cizza G. (2023). Pharmacokinetics, safety, and tolerability of BMS-986263, a lipid nanoparticle containing HSP47 siRNA, in participants with hepatic impairment. Clin. Transl. Sci..

[bb0935] Ramachandran P., Dobie R., Wilson-Kanamori J.R. (2019). Resolving the fibrotic niche of human liver cirrhosis at single-cell level. Nature.

[bb0940] Ramos-Tovar E., Muriel P. (2020). Molecular mechanisms that link oxidative stress, inflammation, and fibrosis in the liver. Antioxidants.

[bb0945] Raza S., Mahamood R., Medhe P. (2025). Sterile inflammation in MASH: emerging role of extracellular RNA and therapeutic strategies. NPJ Metab. Health Dis..

[bb0950] Ren L., Ma X.L., Wang H.L. (2022). Prebiotic-like cyclodextrin assisted silybin on NAFLD through restoring liver and gut homeostasis. J. Control. Release.

[bb0955] Richeldi L., Bois R.M.D., Raghu G. (2014).

[bb0960] Robertson B., Rifkin D.B. (2016). Regulation of the bioavailability of TGF-β and TGF-β-related proteins. Cold Spring Harb. Perspect. Biol..

[bb0965] Robertson B., Horiguchi M., Zilberberg L. (2015). Latent TGF-β-binding proteins. Matrix Biol..

[bb0970] Roderfeld M. (2018). Matrix metalloproteinase functions in hepatic injury and fibrosis. Matrix Biol..

[bb0975] Roeb E. (2018). Matrix metalloproteinases and liver fibrosis (translational aspects). Matrix Biol..

[bb0980] Sahu P., Satapathy T. (2026). Liver targeted nanomedicine for treatment of fibrosis and hepatocellular carcinoma: emerging strategies in ligand-guided, stimuli-responsive and gene-based delivery. J. Drug Delivery Sci. Technol..

[bb0985] Sato T., Head K.Z., Li J. (2023). Fibrosis resolution in the mouse liver: role of Mmp12 and potential role of calpain 1/2. Matrix Biol. Plus.

[bb0990] Schepke M., Wiest R., Flacke S. (2008). Irbesartan plus low-dose propranolol versus low-dose propranolol alone in cirrhosis: a placebo-controlled, double-blind study. Am. J. Gastroenterol..

[bb0995] Schmidt-Arras D., Rose-John S. (2016). IL-6 pathway in the liver: from physiopathology to therapy. J. Hepatol..

[bb1000] Schwabe R.F., Brenner D.A. (2025). Hepatic stellate cells: balancing homeostasis, hepatoprotection and fibrogenesis in health and disease. Nat. Rev. Gastroenterol. Hepatol..

[bb1005] Seitz H.K., Bataller R., Cortez-Pinto H. (2018). Alcoholic liver disease. Nat. Rev. Dis. Primers.

[bb1010] Seki E., Schwabe R.F. (2015). Hepatic inflammation and fibrosis: functional links and key pathways. Hepatology.

[bb1015] Shan L., Wang F., Zhai D. (2023). Matrix metalloproteinases induce extracellular matrix degradation through various pathways to alleviate hepatic fibrosis. Biomed. Pharmacother..

[bb1020] Shang L., Hosseini M., Liu X. (2018). Human hepatic stellate cell isolation and characterization. J. Gastroenterol..

[bb1025] Shen J., Cao J., Chen M. (2023). Recent advances in the role of exosomes in liver fibrosis. J. Gastroenterol. Hepatol..

[bb1030] Shi H., Wang X., Sloas C. (2025).

[bb1035] Shinn J., Park S., Lee S. (2024). Antioxidative hyaluronic acid–bilirubin nanomedicine targeting activated hepatic stellate cells for anti-hepatic-fibrosis therapy. ACS Nano.

[bb1040] Smok-Kalwat J., Mertowska P., Mertowski S. (2023). The importance of the immune system and molecular cell signaling pathways in the pathogenesis and progression of lung cancer. Int. J. Mol. Sci..

[bb1045] Steinberg G., Carpentier A., Wang D. (2025). MASH: the nexus of metabolism, inflammation, and fibrosis. J. Clin. Invest..

[bb1050] Sun M., Kisseleva T. (2015). Reversibility of liver fibrosis. Clin. Res. Hepatol. Gastroenterol..

[bb1055] Sun T., Kang Y., Liu J. (2021). Nanomaterials and hepatic disease: toxicokinetics, disease types, intrinsic mechanisms, liver susceptibility, and influencing factors. J. Nanobiotechnol..

[bb1060] Sun L., Luo X., Zhou C. (2024). Natural polysaccharide-based smart CXCR4-targeted nano-system for magnified liver fibrosis therapy. Chin. Chem. Lett..

[bb1065] Surendran S.P., Thomas R.G., Moon M.J. (2020). A bilirubin-conjugated chitosan nanotheranostics system as a platform for reactive oxygen species stimuli-responsive hepatic fibrosis therapy. Acta Biomater..

[bb1070] Tan Z., Liu Q., Jiang R. (2018). Interleukin-33 drives hepatic fibrosis through activation of hepatic stellate cells. Cell. Molec. Immunol..

[bb1075] Tanaka A., Ma X., Takahashi A. (2024). Primary biliary cholangitis. Lancet.

[bb1080] Tang Z., Li X., Tian L. (2023). Mesoporous polydopamine based biominetic nanodrug ameliorates liver fibrosis via antioxidation and TGF-β/SMADS pathway. Int. J. Biol. Macromol..

[bb1085] Thiele N.D., Wirth J.W., Steins D. (2017). TIMP-1 is upregulated, but not essential in hepatic fibrogenesis and carcinogenesis in mice. Sci. Rep..

[bb1090] Tian T., Zhao C., Li S. (2023). Liver-targeted delivery of small interfering RNA of C–C chemokine receptor 2 with tetrahedral framework nucleic acid attenuates liver cirrhosis. ACS Appl. Mater. Interfaces.

[bb1095] Togami K., Kanehira Y., Nakamura Y. (2025). Pirfenidone encapsulated in succinylated gelatin-coated liposomes exhibits sustained antifibrotic effects in vitro models of renal, pulmonary, and hepatic fibrosis. J. Pharm. Sci..

[bb1100] Tong F., Wang Y., Gao H. (2024). Progress and challenges in the translation of cancer nanomedicines. Curr. Opin. Biotechnol..

[bb1105] Traber P.G., Zomer E. (2013). Therapy of experimental NASH and fibrosis with galectin inhibitors. PLoS One.

[bb1110] Trauner M., Levy C., Tanaka A. (2026). Cilofexor in non-cirrhotic primary sclerosing cholangitis (PRIMIS): a randomised, double-blind, multicentre, placebo-controlled, phase 3 trial. Lancet Gastroenterol. Hepatol..

[bb1115] Trivedi P., Wang S., Friedman S.L. (2021). The power of plasticity—metabolic regulation of hepatic stellate cells. Cell Metab..

[bb1120] Tsuchida T., Friedman S.L. (2017). Mechanisms of hepatic stellate cell activation. Nat. Rev. Gastroenterol. Hepatol..

[bb1125] Tsuchida T., Friedman S.L. (2017). Mechanisms of hepatic stellate cell activation. Nat. Rev. Gastroenterol. Hepatol..

[bb1130] Tung H.-C., Kim J.-W., Zhu J. (2024). Inhibition of heme-thiolate monooxygenase CYP1B1 prevents hepatic stellate cell activation and liver fibrosis by accumulating trehalose.

[bb1135] Vacca M., Kamzolas I., Harder L.M. (2024). An unbiased ranking of murine dietary models based on their proximity to human metabolic dysfunction-associated steatotic liver disease (MASLD). Nat. Metab..

[bb1140] Villesen F., Daniels S.J., Leeming D.J. (2020). The signalling and functional role of the extracellular matrix in the development of liver fibrosis. Aliment. Pharmacol. Ther..

[bb1145] Vitaliti A., Reggio A., Palma A. (2025). Macrophages and autophagy: partners in crime. FEBS J..

[bb1150] Wang X., Wu X., Zhang A. (2016). Targeting the PDGF-B/PDGFR-β interface with destruxin A5 to selectively block PDGF-BB/PDGFR-ββ signaling and attenuate liver fibrosis. EBioMedicine.

[bb1155] Wang Y., Jin G., Li Q. (2016). Hedgehog signaling non-canonical activated by pro-inflammatory cytokines in pancreatic ductal adenocarcinoma. J. Cancer.

[bb1160] Wang K., Zhang Y., Wang G. (2024). FXR agonists for MASH therapy: lessons and perspectives from obeticholic acid. Med. Res. Rev..

[bb1165] Wells R.G. (2013). Tissue mechanics and fibrosis. Biochim. Biophys. Acta.

[bb1170] Wilhelm A., Aldridge V., Haldar D. (2016). CD248/endosialin critically regulates hepatic stellate cell proliferation during chronic liver injury via a PDGF-regulated mechanism. Gut.

[bb1175] Woolbright B.L., Jaeschke H. (2018). Mechanisms of inflammatory liver injury and drug-induced hepatotoxicity. Curr. Pharmacol. Rep..

[bb1180] Xia T., Zhao R., Feng F. (2020). The effect of matrix stiffness on human hepatocyte migration and function—an in vitro research. Polymers.

[bb1185] Xiang L., Wang X., Jiao Q. (2023). Selective inhibition of glycolysis in hepatic stellate cells and suppression of liver fibrogenesis with vitamin A-derivative decorated camptothecin micelles. Acta Biomater..

[bb1190] Xie G., Karaca G., Swiderska-Syn M. (2013). Cross-talk between Notch and Hedgehog regulates hepatic stellate cell fate in mice. Hepatology.

[bb1195] Yamada K.M., Sixt M. (2019). Mechanisms of 3D cell migration. Nat. Rev. Mol. Cell Biol..

[bb1200] Yan J., Huang H., Liu Z. (2020). Hedgehog signaling pathway regulates hexavalent chromium-induced liver fibrosis by activation of hepatic stellate cells. Toxicol. Lett..

[bb1205] Yan Y., Zeng J., Xing L. (2021). Extra- and intra-cellular mechanisms of hepatic stellate cell activation. Biom. J..

[bb1210] Yan M., Cui Y., Xiang Q. (2025). Metabolism of hepatic stellate cells in chronic liver diseases: emerging molecular and therapeutic interventions. Theranostics.

[bb1215] Yang B., Chen Y., Shi J. (2019). Reactive oxygen species (ROS)-based nanomedicine. Chem. Rev..

[bb1220] Yang W., Tao Y., Wu Y. (2019). Neutrophils promote the development of reparative macrophages mediated by ROS to orchestrate liver repair. Nat. Commun..

[bb1235] Yang J., Liang Z., Zhao H. (2025). Nanoparticle-mediated liver targeted delivery of IFN-γ confers enhanced anti-fibrotic efficacy with reduced systemic toxicity. Int. J. Biol. Macromol..

[bb1240] Yao L., Li J., Qin X. (2023). Antifibrotic and antioxidant effects of a tetrahedral framework nucleic acid-based chlorogenic acid delivery system. ACS Mater. Lett..

[bb1245] Yaramiri A., Asalh R.A., Asalh M.A. (2025). A comprehensive review of smart thermosensitive nanocarriers for precision cancer therapy. Int. J. Mol. Sci..

[bb1250] Ye Z., Cheng K., Guntaka R.V. (2006). Receptor-mediated hepatic uptake of M6P-BSA-conjugated triplex-forming oligonucleotides in rats. Bioconjug. Chem..

[bb1255] Yoshida K., Matsuzaki K., Murata M. (2018). Clinico-pathological importance of TGF-β/phospho-smad signaling during human hepatic fibrocarcinogenesis. Cancers.

[bb1260] You D.G., Oh B.H., Nguyen V.Q. (2021). Vitamin A-coupled stem cell-derived extracellular vesicles regulate the fibrotic cascade by targeting activated hepatic stellate cells in vivo. J. Control. Release.

[bb1265] Younis M.A., Sato Y., Elewa Y.H.A. (2023). Reprogramming activated hepatic stellate cells by siRNA-loaded nanocarriers reverses liver fibrosis in mice. J. Control. Release.

[bb1270] Yuan Y., Li J., Chen M. (2024). Nano-encapsulation of drugs to target hepatic stellate cells: toward precision treatments of liver fibrosis. J. Control. Release.

[bb1275] Zhang Y.E. (2017). Non-Smad signaling pathways of the TGF-β family. Cold Spring Harb. Perspect. Biol..

[bb1280] Zhang Z., Zhao S., Yao Z. (2017). Autophagy regulates turnover of lipid droplets via ROS-dependent Rab25 activation in hepatic stellate cell. Redox Biol..

[bb1285] Zhang C., An R., Bao Y.-W. (2019). Inhibitory effects of octreotide on the progression of hepatic fibrosis via the regulation of Bcl-2/Bax and PI3K/AKT signaling pathways. Int. Immunopharmacol..

[bb1290] Zhang J., Shen H., Xu J. (2020). Liver-targeted siRNA lipid nanoparticles treat hepatic cirrhosis by dual antifibrotic and anti-inflammatory activities. ACS Nano.

[bb1295] Zhang M., Serna-Salas S., Damba T. (2021). Hepatic stellate cell senescence in liver fibrosis: characteristics, mechanisms and perspectives. Mech. Ageing Dev..

[bb1300] Zhang Z., Wang X., Wang Z. (2021). Dihydroartemisinin alleviates hepatic fibrosis through inducing ferroptosis in hepatic stellate cells. Biofactors.

[bb1305] Zhang H., Dong X., Zhu L. (2024). Elafibranor: a promising treatment for alcoholic liver disease, metabolic-associated fatty liver disease, and cholestatic liver disease. World J. Gastroenterol..

[bb1310] Zhang J., Zhang T., Zhang Z. (2024). The dual-target nanoparticles with ROS sensitivity inhibit the hedgehog signaling pathway and decrease oxidative stress in activated hepatic stellate cells to alleviate liver fibrosis. Adv. Funct. Mater..

[bb1315] Zhang Y., Ren L., Tian Y. (2024). Signaling pathways that activate hepatic stellate cells during liver fibrosis. Front. Med..

[bb1320] Zhang J., Chen S., Zhou J. (2025). Serial liver stiffness measurement and serum biomarkers are not strong predictors of the regression of fibrosis among chronic hepatitis B patients receiving antiviral therapy based on triple liver biopsies. Gut Liver.

[bb1325] Zhang J., Xie Z., Zhu X. (2025). New insights into therapeutic strategies for targeting hepatic macrophages to alleviate liver fibrosis. Int. Immunopharmacol..

[bb1330] Zhang H., Xing J., Sun M. (2026). Engineered exosomes for targeted microRNA delivery to reverse liver fibrosis. Biom. J..

[bb1335] Zhang L., Liu T., Shen H. (2026). Advanced strategies based on nanomedicine for liver fibrosis treatment. Int. J. Pharm..

[bb1340] Zhao X., Amevor F.K., Xue X. (2023). Remodeling the hepatic fibrotic microenvironment with emerging nanotherapeutics: a comprehensive review. J. Nanobiotechnol..

[bb1345] Zhao K., Chan T.C., Tse E.H.Y. (2025). Autophagy in adult stem cell homeostasis, aging, and disease therapy. Cell Regener..

[bb1350] Zhao Y.X., Sun Y.Y., Li L.Y. (2025). Rab11b promotes M1-like macrophage polarization by restraining autophagic degradation of NLRP3 in alcohol-associated liver disease. Acta Pharmacol. Sin..

[bb1355] Zhao C., Xiao A., Chen C. (2026). Nanotechnology for diagnosis and therapy of idiopathic pulmonary fibrosis: recent advances and future perspectives. Nano Today.

[bb1360] Zhou J.E., Sun L., Liu L. (2022). Hepatic macrophage targeted siRNA lipid nanoparticles treat non-alcoholic steatohepatitis. J. Control. Release.

[bb1365] Zhou L., Liang Q., Li Y. (2022). Collagenase-I decorated co-delivery micelles potentiate extracellular matrix degradation and hepatic stellate cell targeting for liver fibrosis therapy. Acta Biomater..

[bb1370] Zuo T., Xie Q., Liu J. (2023). Macrophage-derived cathepsin S remodels the extracellular matrix to promote liver fibrogenesis. Gastroenterology.

